# E2F1-autophagy-ALDH1A1 axis enhances self-renewal and drug resistance of lung cancer stem-like cells in a p53-dependent manner

**DOI:** 10.1186/s13046-025-03506-4

**Published:** 2025-08-30

**Authors:** Jingyuan Li, Yiyu Chen, Jianyu Wang, Liyuan Liu, Javeria Qadir, Dan Xie, Xue Wan, Yanan Luo, Jiawen Xian, Ting Ye

**Affiliations:** 1 Department of Laboratory Medicine, the Affiliated Hospital of Southwest Medical University, No. 25, Taiping Street, Jiangyang District, Luzhou, Sichuan 646000 P. R. China; 2 Molecular Biology Laboratory of Respiratory Disease, Key Laboratory of Clinical Laboratory Diagnostics (Ministry of Education), College of Laboratory Medicine, Chongqing Medical University, Chongqing, 400016 P. R. China; 3 Department of Biosciences, COMSATS University Islamabad, Islamabad, 44000 Pakistan

**Keywords:** Lung cancer stem cells, E2F1, Autophagy, p53, Self-renewal, Drug resistance

## Abstract

Lung adenocarcinoma (LUAD) is a predominant subtype of non-small cell lung adenocarcinoma (NSCLC). It is typically asymptomatic and associated with high mortality rates. Despite recent advancements in screening technologies and therapeutic approaches, its pathogenesis still remains elusive. Therefore, it is imperative to explore new diagnostic markers and therapeutic targets for LUAD management. Cancer stem cells (CSCs) have high self-renewal capacity and incur therapeutic resistance, thus, considered as crucial elements in initiating and promoting tumor development. Contextual to this, the present study reveals the role of the transcriptional activator E2F1 in LUAD oncogenesis and its association with various biological characteristics of lung cancer stem cells (LCSCs). Whereby, it may also serve as a crucial factor in regulating autophagy. Autophagy can modulate stemness by either promoting or inhibiting CSCs characteristics. Pertinently, our study integrated bioinformatics, in-vitro and in-vivo experiments to elucidate that E2F1 can induce ALDH1A1 through autophagy, thus promoting self-renewal and drug resistance of LCSCs, as well as tumorigenicity. Mechanistically, “E2F1-autophagy-ALDH1A1” axis enhanced the self-renewal capacity and drug resistance of LCSCs in a p53-dependent manner, highlighting the potential of E2F1 as a promising marker for LUAD.

**Supplementary Information:**

The online version contains supplementary material available at 10.1186/s13046-025-03506-4.

## Highlights

- High self-renewal, drug resistance, and autophagy flux of LCSCs.
- High-level E2F1 promoting autophagy to strengthen self-renewal and drug resistance of LCSCs.
- The “E2F1-autophagy-ALDH1A1” axis regulates self-renewal and drug resistance of LCSCs in a p53-dependent manner.
- The transcription factor E2F1 as a potential biomarker for LUAD.

**Supplementary Information:**

The online version contains supplementary material available at 10.1186/s13046-025-03506-4.

## Introduction

Non-small cell lung cancer (NSCLC) is the main histological subtype of lung cancer. Of which, lung adenocarcinoma (LUAD) is the most pre-dominant subtype of NSCLC. Due to its asymptomatic nature, metastatic potential and therapeutic resistance, the overall 5-year survival rate is less than 20% in LUAD [1, 2]. Correspondingly, cancer stem cells (CSCs) are tumor cells with therapeutic resistance, oncogenicity and stemness characteristics, which are considered to have critical role in mediating tumor initiation and progression [3, 4]. Previous reports have also established the importance of CSCs renewal in sustaining tumor progression [5, 6]. The mechanisms of CSCs stemness are complicated, and include abnormal activation of pluripotency genes or signal transduction pathways [7], non-coding RNAs [8], and metabolism [9]. Therefore, revealing the crucial genes and intrinsic mechanisms behind CSC stemness is important for targeting CSCs and scheming anti-tumor therapy.

The E2F family of transcription factors (E2Fs) was originally identified as transcriptional activators of the adenovirus E2 gene promoter, which interact with retinoblastoma protein (Rb), thus participating in the regulation of diverse biological processes such as cell cycle, apoptosis, and DNA replication [10, 11]. E2Fs are classified as transcriptional activators (E2F1-E2F3a) and transcriptional repressors (E2F3b-E2F8) [12]. Among these, E2F1 is the first member of the E2Fs to be discovered that could bind both to Rb to exhibit tumor-suppressive effects [13], and induce tumorigenesis, drug resistance, and other adverse outcomes through its role as a transcriptional activator [14, 15]. E2F1 does not only act as an oncogene promoting stemness in ovarian, gastric, and laryngeal squamous cell carcinoma CSCs [16–18], but also interacts with the long non-coding RNA DLGAP1-AS2, upregulating CD151 expression, thus increasing resistance to radiotherapy in colorectal cancer stem cells [19]. Moreover, previous studies suggest E2F1 as an autophagy regulator that could potentially enhance autophagy by directly transcribing microtubule-associated protein 1 light chain 3 beta (MAP1LC3/LC3B), autophagy-related gene-1 (ATG1), and damage-regulated autophagy modulator (DRAM) or indirectly regulating ATG5 [20]. Conversely, E2F1 also functions as a suppressor in hepatocellular carcinoma, interacting with ubiquitin specific peptidase 11 (USP11) to regulate the extracellular-signal regulated protein kinase/mammalian target of Rapamycin (ERK/mTOR) pathway to inhibit autophagy [21]. Similarly, several studies have observed that E2F1 regulates autophagy in a dual manner i.e., it acts both as a promoter and an inhibitor, whereby the function of E2F1 in LCSCs is urgently required to be further elucidated. Therefore, this study aims to investigate the function and underlying regulatory mechanism of E2F1 in LCSCs, thereby, assisting in the early diagnosis and treatment of LUAD.

Autophagy is an essential metabolic program which involves the degradation of damaged transporting proteins and organelles by lysosomes. It is crucial for maintaining cellular homeostasis, proliferation, and disease prevention [22]. Autophagy is classified into macro-autophagy/autophagy, micro-autophagy, and chaperone-mediated autophagy (CMA), with autophagy being the most extensively studied [23]. These studies have shown that defects or abnormal activation of autophagy can have significant implications for several diseases such as senescence [24], neurodegenerative disorders [25]and cancer [26]. While autophagy has dual role in regulating the biological characteristics of CSCs, some studies are conducive to CSC stemness [27, 28]. Contrary to this, others have proposed that overstimulation of autophagy inhibits the stemness of CSCs [29]. Hence, we explored if the expression and functions of autophagy in LCSCs are significantly altered to provide insights into LUAD diagnosis and its treatment. Furthermore, subcellular localization of p53, which regulates various cell death pathways including apoptosis, autophagy and necroptosis has been deciphered [30]. Tasdemir E [31] demonstrated that cytoplasmic p53 inhibits autophagy by AMPK, whereas, nuclear localized p53 regulates mTOR signaling in a transcription-dependent manner to induce autophagy [32].

The aldehyde dehydrogenase family (ALDH), is a group of endogenous enzymes that impart stem cell properties and serve as a marker for CSCs. It is constituted of 19 isoenzymes with prominent physiological and toxicological functions. Among them, aldehyde dehydrogenase 1A1 (ALDH1A1) regulates the characteristics of CSCs, metabolism and DNA repair, thereby exerting its pro-angiogenic functions, promoting metastasis, and drug resistance [33]. Additionally, ALDH1A has been shown to be an independent prognostic biomarker in LUAD, and if combined with CD133, it serves as a prognostic indicator in NSCLC [34]. Otherwise, ALDH1A1 can also confer resistance to erlotinib in LUAD by promoting reactive oxygen species-reactive carbonyl species (ROS-RCS) metabolic regulation [35]. Therefore, we could elucidate that the biological function of ALDH1A1 is important in investigating oncogenesis and novel therapeutics.

In the present study, we generated human LCSC cell lines and demonstrated their characteristics of high-level autophagic flux, self-renewal and drug resistance. It was further revealed that transcription factor E2F1 is upregulated in LUAD and its CSCs. This study also demonstrates the p53-dependent mechanism by which E2F1 enhances autophagy to promote self-renewal and drug resistance of LCSCs. Finally, our study validated E2F1 as a potential biomarker for LUAD management.

## Materials and methods

### Cell lines and cell culture

A549 and H1299 parental cells were cultured in RPMI 1640 culture medium, containing 1% penicillin-streptomycin-amphotericin B (C100C8, New Cell & Molecular Biotech) and 10% FBS (Gibco, USA). A549 and H1299 spheroid cells were cultured in DMEM/F12 (1:1) culture medium, supplemented with 2% B27 (Gibco, USA), 1% penicillin-streptomycin-amphotericin B, 20ng bFGF (Beyotime) and 20ng EGF (Beyotime). All the cell lines were cultured and maintained in the incubator at 37 °C with 5% CO_2_ atmosphere.

### Total RNA extraction and real time quantitative PCR (RT-qPCR)

Total RNA was isolated using Trizol reagent (Takara, Japan), and reverse-transcribed using Prime Script™ RT Master Mix (Takara, Japan) in accordance with the manufacturer’s protocol. RT-qPCR was performed with TB Green™ Premix Ex Taq™ kit (Takara, Japan) using Bio-Rad qPCR system. The mRNA expression was normalized to TBP and calculated using the formula 2^−ΔΔCt^. The sequences of PCR primers are provided in the Table S1.

### Western blot

Cells were lysed in RIPA lysis buffer with 1% PMSF (Beyotime). Total protein concentration was measured by BCA protein quantitative kit (WB6501, New Cell & Molecular Biotech), and the final concentration was adjusted to 25 µg. Each sample was resolved by 10% or 15% SDS-PAGE gels and transferred to PVDF membrane (Bio-Rad, USA). The membrane was blocked in 5% skimmed milk (BD, USA) and immunoblotted with primary antibodies at 4 °C overnight. Then, the membrane was incubated with secondary antibodies at room temperature for 1 h. Finally, the blots were visualized by ECL solution (Beyotime, P0018HM), scanned by gel electrophoresis imager (Bio-Rad, USA). The primary antibodies: p53 (10442-1-AP, 1:2000), E2F1 (66515-1-Ig, 1:2000), LC3B (14600-1-AP, 1:1500), p62 (66184-1-Ig, 1:2000), ATG5 (10181-2-AP, 1:2000), Beclin1 (11306-1-AP, 1:5000), GAPDH (60004-1-Ig, 1:10000), ALDH1A1 (60171-1-Ig, 1:1000). The secondary antibodies: HRP-conjugated Affinipure Goat Anti-Mouse IgG IgG(H + L) (SA00001-1, 1:7500), HRP-conjugated Affinipure Goat Anti-Rabbit IgG IgG(H + L) (SA00001-1,1:7500). All of the above antibodies were purchased form Proteintech (USA).

### Flow cytometry

Cells were collected and washed twice utilizing PBS, followed by experiments according to the Annexin V-FITC/PI Apoptosis Kit (Beyotime, C1062) and ALDH1A1 instructions, respectively. In addition, detection of ALDH1A1 expression requires fixation by pre-cooled 4% paraformaldehyde, rupture of cell membranes by pre-cooled 4% paraformaldehyde fixation and 90% methanol, and addition of ALDH1A1 primary antibody and FITC-labeled fluorescent secondary antibody, sequentially. Finally, 200–500 µl of PBS was used to resuspend the cells. To ensure accurate fluorescence detection and to account for spectral overlap between different fluorochromes, compensation adjustments were performed. Each sample mentioned above was measured by BD CytoFLEX flow cytometry.

### 96-well plate single clone assay

A single-cell suspension was prepared and the concentration was single cell/well with 100 µl of culture medium. The 96-well plates were viewed under the microscope and the wells containing a single cell were marked. After 7–10 days of culture at 37 °C with 5% CO_2_, the colonies grown were observed and counted under the microscope.

### Cell counting kit 8 (CCK8) assay

Cells were inoculated in 96-well plates at a density of 2 × 10^3^ and cultured at 37 °C with 5% CO_2_. After treating the cells with cisplatin for 24 h, cell viability was assessed according to the instructions of the CCK8 kit (Beyotime, C0039), and the absorbance of the cells at 450 nm was determined by placing the 96-well plates in an enzyme-linked immunoassay instrument.

### Subcutaneous tumor transplantation in balb/c nude mice

6–8 weeks old BALB/c nude mice were purchased from Beijing HFK Bio-Technology.co. The cells were collected by centrifugation, counted after resuspension using PBS, mixed with stromal gel (R&D System, USA) at 1:1, and injected subcutaneously into the bilateral groin of nude mice at 10^2^, 10^3^, and 10^4^ cells/side respectively to validate the gradient oncogenesis of CSCs. Next, the cells were inoculated on each side of the nude mice subcutaneously to confirm the tumorigenicity of E2F1. Tumor formation was observed, and the tumor volume was measured at 1-day intervals. The formula used for calculating the tumor volume was V = 1/2×length×width×width. The nude mice were then euthanized when the tumor volume reached 1 cm^3^, and the removed tumor tissues were weighed. All animal experiments were approved and licensed by the Animal Ethics Committee of Southwest Medical University (NO. 20211121-003).

### Immunofluorescence staining analysis

Cells were seeded into cells slides in six-well plates with 5 × 10^4^ cells for immunofluorescence staining. The slides were washed twice with PBS, fixed for 30 min with 4% paraformaldehyde. After washed three times with PBS, Triton X-100 (Beyotime, P0096) for 5 min, and then blocked in goat serum for 1 h at room temperature, the slides were then incubated overnight with anti-LC3 antibody (Proteintech, 14600-1-AP, 1:500 dilution) at 4°C. The following secondary antibodies were used: goat anti-rabbit IgG/Alexa Fluor/488 (ZF-0511, 1:200 dilution) for 1 h and stained with DAPI (Beyotime, C1005) for 10 min at room temperature. The cells slides were observed and photographed using a microscope (OLYMPUS, Japan). This study was approved by the Institutional Review Board of the Affiliated Hospital of Southwest Medical University (NO.KY2024460), and informed consent was waived due to the retrospective nature of the research.

### Autophagy-flux detection

Cells were inoculated in confocal petri dishes at a density of 5 × 10^4^ cells per dish, and transfection was performed according to the instructions of the Ad-mCherry-GFP-LC3B kit (Beyotime, C3011) when the cell growth density was about 50%. Using a laser confocal microscope (Leica, Japan), the yellow spot form (LC3B dot or puncta) was observed microscopically and images were captured.

### Transmission electron microscope (TEM)

Cells were inoculated into 60 mm dishes and collected by centrifugation at 1500 rpm/min for 10 min. The cells were resuspended dropwise with approximately 0.5% glutaraldehyde fixative and allowed to stand for 10 min at 4 °C. The supernatant was discarded by centrifugation at 12,000 rpm/min for 15 min, and the precipitate was retained. 3% glutaraldehyde fixative was slowly added along the wall of the tube and sent to the laboratory (Chengdu Lilai Biomedicine Co., Ltd).

### Cell transient transfection

Cells were inoculated into 6-well plates at a density of 5 × 10^4^ cells/well and cultured for 24 h. The transfection system of plasmid and short interfering RNA (siRNA) was prepared according to the instructions of Lipo8000™ Transfection Reagent (Beyotime, C0533). The cells were incubated at 37 °C with 5% CO_2_ for 72 h. The si-Beclin1 sequence is shown in Table S2.

### Construction of a cancer stem cell-like properties assessment model (stemness score)

To construct the assessment model, literature from PubMed and data from Stem Checker (http://stemchecker.sysbiolab.eu/) were used to select stemness-related gene sets reflecting stem cell gene expression. Two gene chips from the GEO database (http://www.ncbi.nlm.nih.gov/geo,GSE30654 and GSE34200) provided the basis for constructing the stem cell gene sets. ssGSEA calculated stemness scores for each sample in the 28 selected gene sets. ROC curves evaluated the gene sets’ ability to distinguish stem cells from somatic cells, excluding those with AUC < 95%. This resulted in 13 gene sets with higher scores in tumor stemness-like cells. The remaining sets were used to calculate enrichment scores in 1019 tumor cell lines from the CCLE (Cancer Cell Line Encyclopedia, https://sites.broadinstitute.org/ccle/). Gene sets with CV > 0.1 were excluded, leaving 11 stemness-related sets. Using TCGA data (https://portal.gdc.cancer.gov/) from 32 cancers, correlations of the final gene sets were analyzed, selecting 214 genes for the final stemness-specific set to assess stemness scores in tissues or cells.

### Verification of stemness score model in TCGA database

LUAD data with different stemness scores were divided into high and low groups, and the expression levels of stemness markers were confirmed between these groups. The levels of stemness scores were compared between lung cancer tissues and normal lung tissues, lung cancer tissues and corresponding adjacent tissues, early and advanced stage patients, as well as metastatic and non-metastatic LUAD. Furthermore, receiver operating characteristic (ROC) curves, Kaplan-Meier survival analysis, and Cox regression analysis were performed to demonstrate the diagnostic and prognostic efficacy of the stemness score.

### Gene set enrichment analysis of stemness scores

Gene Set Enrichment Analysis (GSEA) was performed to annotate the Hallmark effector gene sets and the Kyoto Encyclopedia of Genes and Genomes (KEGG) signaling pathways associated with stemness scores in the TCGA-LUAD dataset. The GSEA software was obtained from the Broad Institute (http://www.broad.mit.edu/gsea).

### GO functional enrichment analysis

Aberrantly expressed genes were filtered using transcription profiles from the TCGA-LUAD database. The correlation coefficients were calculated using Pearson correlation to identify genes related to stemness scores among differentially expressed genes (*r* > 0.4, *P* < 0.05). Subsequently, bioinformatic analysis of the GO Enrichment analysis was performed using R software and Bioconductor packages.

### Survival analysis of overall survival (OS) in TCGA database

Gene expression in LUAD was analyzed by TCGA Research Network (http://cancergenome.nih.gov). The original data from the TCGA database was normalized and analyzed by the edgeR analysis method. To analyze the overall survival of patients with LUAD, patient samples were analyzed by Kaplan-Meier analysis.

### Analysis of the ATAC-seq in TCGA database

The ATAC-seq data were obtained from primary breast cancer tissue samples collected by TCGA. These data were used to cluster the samples and identify epigenetically defined patient subgroups. In addition, using the ATAC-seq data, the enrichment of cancer-promoting gene promoter regions and cancer-related functions were also identified.

### Lentivirus transfection

Lentivirus was transfected to construct A549/H1299 spheroid cell lines, and the corresponding negative control cell line was established by infecting with an empty load of virus. Collected cells were inoculated at 5 × 10^4^ in 24-well plates and cultured for 24 h according to the instructions. Subsequently, they were incubated at 37 °C in 5% CO_2_ atmosphere for 72 h.

### Immunohistochemistry (IHC)

Tissue slides were fixed at 56 °C for at least 2 h, deparaffinized using xylene and then hydrated through an ethanol gradient. Subsequently, heat-induced epitope repair was performed using sodium citrate, and endogenous enzymes were removed by adding 3% hydrogen peroxide. Then, the slides were blocked using goat serum (CW0130S, CWBIO) for 1 h at room temperature and incubated overnight at 4 °C with primary antibody. The next day, the tissue slides were equilibrated at room temperature for 30 min and then washed, and the tissue slides were processed according to the instructions of SABC IHC Kit (Biosharp, BL733A). The tissues were visualized using the DAB kit (ZSBio, ZLI-9018), and hematoxylin re-stained and neutral resin-sealed slices were placed under a microscope for visualization and imaging.

### Enzyme-linked immunosorbent assay (ELISA)

Human E2F1 (transcription factor E2F1) ELISA Kit (Fine Biotech, China) was used to detect the protein expression of E2F1 in the serum of healthy people and lung adenocarcinoma patients according to the instructions provided with the kit. The serum expression level was determined by measuring the absorbance optical density (OD) at 450 nm, and a standard curve was established with OD450 as the Y-axis and the concentration of the standard as the X-axis.

### Statistical analysis

Statistical analyses were performed by Student’s independent t-test and one-way ANOVA using GraphPad Prism 8 software (GraphPad Software). Data were expressed as mean ± standard deviation (SD), *P* < 0.05 was considered statistically significant. All the *P* values are labeled in the graphs (*P < 0.05; **P < 0.01; ***P < 0.001), and all the experiments were repeated at least three times.

## Results

### The characteristics of high stemness, drug resistance and autophagy level in LCSCs

**Fig. 1:**
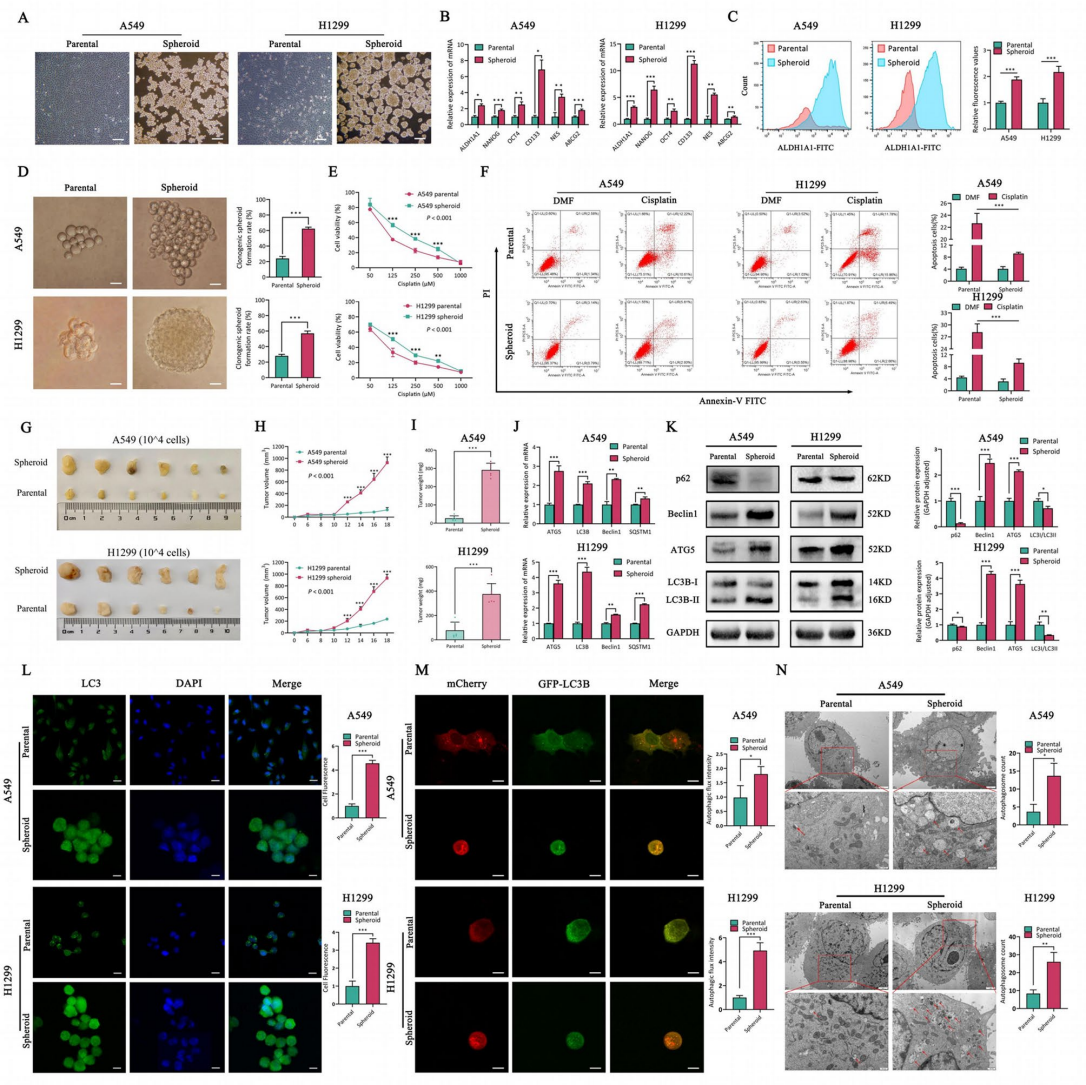
Lung cancer stem cells exhibit high stemness, drug resistance, and autophagic fluxes. (**A**) The morphology of LUAD parental and spheroid cells, scale bar = 30 μm. (**B**) Expression of stem genes in parental and spheroid cells of LUAD by RT-qPCR, TBP as the internal control. (**C**) Flow cytometry analysis of ALDH1A1 expression in LUAD parental and spheroid cells. The Right panel represents quantification of ALDH1A1 expression. (**D**) Spheroid formation in LUAD parental and spheroid cells by single clone assay. The Right panel represents quantification of single clone assay. (**E**) Proliferation activities of LUAD parental and spheroid cells incubated with cisplatin by CCK8. (**F**) The effect of cisplatin on apoptosis in LUAD parental and spheroid cells by flow cytometry. The Right panel represents quantification of apoptosis cells. (**G**) Tumor bearing nude mice were inoculated with LUAD parental and spheroid cells (1 × 10^4^ cells), respectively. **H-I**. Tumor growth curves of LUAD parental and spheroid cells in nude mice. (**H**) Tumor volume. (**I**) Tumor weight. (**J**) The mRNA expression of autophagy related genes in parental and spheroid cells of LUAD by RT-qPCR, TBP as the internal control. **K**. The protein levels of p62, Beclin1, ATG5, LC3B in LUAD parental and spheroid cells assessed by Western blotting, GAPDH protein was used as the internal control. The Right panel represents quantification. **L**. Immunofluorescent staining of LC3B protein in LUAD parental and spheroid cells, scale bar = 30 μm. The Right panel represents quantification of fluorescence expression. **M**. LUAD parental and spheroid cells were transfected with GFP-mRFP-LC3 to analyze LC3 puncta by confocal microscope, scale bar = 30 μm. The Right panel represents quantification of autophagic flux intensity. **N**. Transmission electron microscopy analyzed the amount of autophagic structures (indicated by red arrow), scale bar = 2 μm (upper panels), scale bar = 500 nm (lower panels). The Right panel represents quantification of autophagic structures. *P < 0.05, **P < 0.01, ***P < 0.001

We firstly isolated and purified LCSCs from LUAD parental cells [36] (Fig. 1A). Next, we confirmed higher self-renewal characteristics of LCSCs by pluripotency genes expression and cell’s ability to form monoclonal colonies (Fig. 1B-D). In addition, to assess the therapeutic resistance traits of CSCs, we treated LUAD and its CSCs with cisplatin, verifying LCSCs to confer increased drug resistance (Fig. 1E-F). Moreover, 10^4^ LUAD cells and its CSCs were injected subcutaneously in the nude mice to validate in vivo oncogenicity (Fig. 1G-I). The above results indicated that LCSCs possess self-renewal, drug resistance and tumorigenicity. Then, we revealed the autophagy activity in LCSCs through: (1) the expression of the autophagy related genes and proteins expression (Fig. 1J-K) (2), the expression of LC3B by immunofluorescence (Fig. 1L) (3), LC3B puncta by confocal microscopy (Fig. 1M), and (4) TEM analysis of autophagosomes (Fig. 1N). Together, we indicated that LCSCs augment autophagic activity compared to the LUAD parental cells.

### Autophagy augments stemness and drug resistance in A549 spheroid cells, but not the H1299 spheroid cells

**Fig. 2:**
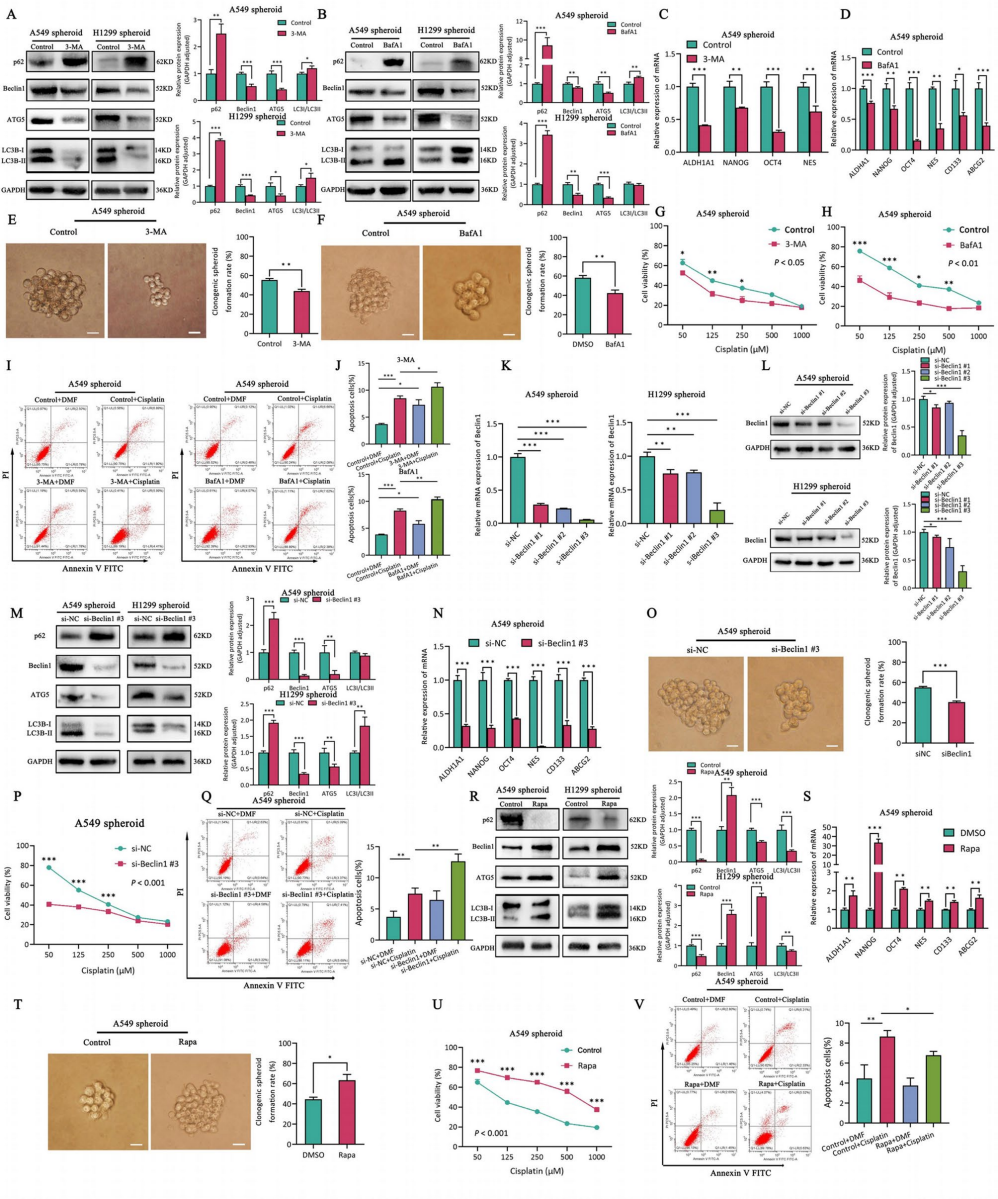
Autophagy enhances self-renewal and drug resistance of lung cancer stem cells. **A-B**. The levels of autophagy related protein in LUAD spheroid cells incubated with autophagy inhibitor 3-MA (**A**) or autophagy inhibitor BafA1 (**B**) compared with the control cells, GAPDH protein was used as the internal control. The Right panel represents quantification. **C-D**. The expression of CSCs marker in LUAD spheroid cells incubated with 3-MA (**C**) or BafA1 (**D**) compared with the control cells, TBP as the internal control. **E-F**. Spheroid formation in LUAD parental and spheroid cells incubated with autophagy inhibitor 3-MA (**E**) or BafA1 (**F**) and control by single clone assay. The Right panel represents quantification of single clone assay. **G-H**. The cell viability of LUAD spheroid cells incubated with 3-MA (**G**) or BafA1 (**H**) and control cells was detected by CCK8 assay. **I-J**. Flow cytometric analysis of apoptosis in A549 spheroids treated with cisplatin, with or without the addition of 3-MA or BafA1. Cells were stained with Annexin V-FITC and propidium iodide (PI) to assess apoptosis (**I**). The Right panel represents quantification of apoptosis cells (**J**). **K-L**. The expression of Beclin1 in si-NC and si-Beclin1 of LUAD spheroid cells, si-NC was the negative control, si-Beclin1 knocked down Beclin1 by transfecting with siRNA. K. RT-qPCR. **L**. Western blotting, The Right panel represents quantification. **M**. Autophagy relative protein abundance in si-NC and si-Beclin1 of LUAD spheroid cells. The Right panel represents quantification. **N-O**. The self-renewal capability of si-NC and si-Beclin1 LUAD spheroid cells. **N**. mRNA abundance of stemness genes. **O**. Single clone assay. The Right panel represents quantification of single clone assay. **P-Q**. The therapy resistance of si-NC and si-Beclin1 LUAD spheroid cells. **P**. CCK8. **Q**. Flow cytometry. The Right panel represents quantification of apoptosis cells. **R**. The protein levels in LUAD spheroid cells treated with autophagy activator Rapamycin and control. The Right panel represents quantification. **S-T**. The self-renewal capability of LUAD spheroid cells treated with Rapa and control. **S**. RT-qPCR. **T**. Single clone assay. The Right panel represents quantification of single clone assay. **U-V**. Drug resistance in LUAD spheroid cells treated with Rapa and control. **U**. CCK8. **V**. Flow cytometry. The Right panel represents quantification of apoptosis cells. *P < 0.05, **P < 0.01, ***P < 0.001

In order to clarify the function of autophagy in LCSCs, firstly, autophagy was suppressed by 3-Methyladenine (3-MA) (Fig. 2A) and Bafilomycin A1 (BafA1) (Fig. 2B), which inhibits the early- and late-stage of autophagy program, respectively. We demonstrated a decline in pluripotency genes expression (Fig. 2C-D), and single clone formation (Fig. 2E-F) also showed that self-renewal of CSCs was inhibited in A549 spheroid cells. Meanwhile, the results also indicated that the cisplatin resistant activity of A549 spheroid cells was reduced (Fig. 2G-H, I-J). However, no significant changes in self-renewal and drug tolerance were observed in H1299 spheroid cells (Fig.S1A-H). Thereafter, we silenced autophagy gene Beclin1 by siRNA to inhibit autophagy (Fig. 2K-M). Similarly, it was observed that the knockdown of Beclin1 significantly reduced self-renewal (Fig. 2N-O) and drug resistance (Fig. 2P-Q) of A549 spheroid cells, but had no effect on H1299 spheroid cells (Fig.S1I-J, S1M, S1O, S1Q). As well, the autophagy activator Rapamycin (Rapa) elevated the autophagy activity of A549 and H1299 spheroid cells (Fig. 2R), as well as enhanced self-renewal (Fig. 2S-T) and cisplatin tolerance in A549 spheroid cells (Fig. 2U-V), while the properties were not observed in H1299 spheroid cells (Fig.S1K-L, S1N, S1P, S1Q).

### Bioinformatic analysis identified E2F1 as a key regulator of stemness and autophagy in LUAD

**Fig. 3:**
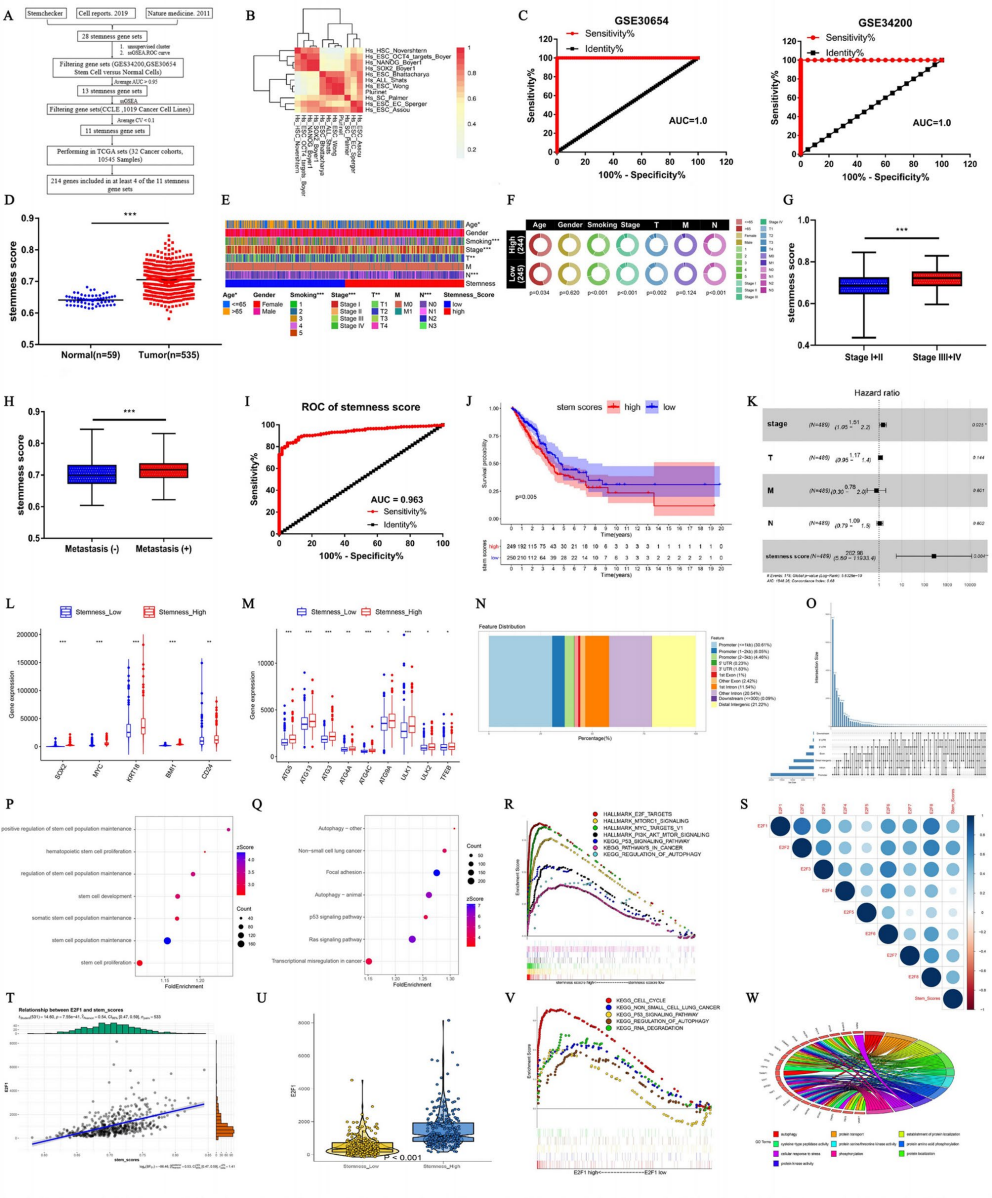
E2F1 is screened as a key regulator of stemness and autophagy in LUAD based on the “stemness score” model. (**A**) A flow chart indicating stemness genes screening. (**B**) Correlation heat map of enrichment scores of 11 gene sets in TCGA-LUAD samples. (**C**) Analyzing the reliability of stemness scores based on GEO database (GSE30654, GSE342000). (**D**) The stemness scores level in TCGA-LUAD dataset. **E-F**. The heat map (**E**) and pie map (**F**) demonstrate the value of stemness scores in evaluating LUAD-related clinical parameters. **G-H**. Comparison of stemness scores in different clinical parameter LUAD, Tumor staging (**G**), Tumor metastasis (**H**). **I**. The AUC of stemness score in TCGA-LUAD dataset. **J**. Kaplan-Meier estimates of the OS of patients with LUAD according to stemness score based on TCGA-LUAD dataset. **K**. Multivariate analysis of the correlation of stemness score with LUAD patients. **L-M**. The stemness-related (**L**) and autophagy-related (**M**) gene expression of stemness score high and low groups. **N**. Bar graph showing the enriched proportion of stemness-high peaks. **O**. Chart comparing the distribution of promoter area. **P**. Enrichment GO analysis showing the stemness function of peaks. **Q**. Enrichment KEGG analysis showing the potential function of peaks. **R**. GSEA assessment of stemness score high and low groups. **S**. Correlation of the expression of E2F family and stemness scores in LUAD. **T**. Correlation of the expression of E2F1 and stemness scores. U. Abundance of E2F1 mRNA expression of stemness score high and low groups. **V**. KEGG analysis of E2F1 high and low groups. **W**. GO enrichment analysis of E2F1 high and low groups. *P < 0.05, **P < 0.01, ***P < 0.001

The computational method established to assess the enrichment degree of stemness-like cells from tumor tissue transcriptome data was evaluated using TCGA whole transcriptome data (Fig. 3A). It was found that the final inclusion of 11 gene sets for evaluating tumor enrichment patterns exhibited high consistency (Fig. 3B). After integrating the 11 gene sets into the final stem cell-specific gene set, 214 genes present in at least 4 of the gene sets were selected and defined as the stemness scoring set. This gene set was used to validate two groups of GEO datasets containing stem and somatic cells, revealing that the final specific gene set nearly perfectly distinguished between these two cell types (Fig. 3C). The stemness-like cell enrichment degree (stemness scores) was used to assess the enrichment of stem-like cells in each tissue of lung adenocarcinoma, confirming that these scores are significantly higher than in adjacent non-cancerous tissues (Fig. 3D). Further analysis of the patient’s clinical data revealed significant differences in stemness scores across age, number of cigarettes smoked, stage, and lymph node metastasis (Fig. 3E-F). The stemness score is significantly higher in patients with advanced-stage cancer compared to those in early stages (Fig. 3G), and in patients with metastases compared to those with no metastasis (Fig. 3H). Analysis of the relationship between the stemness scores, and the diagnosis and prognosis of LUAD patients confirmed a high diagnostic efficiency (Fig. 3I, AUC = 0.963). The stemness scores were not only negatively related to the prognosis of LUAD but also served as an independent prognostic factor for these patients (Fig. 3J-K). Further analysis revealed that LUAD cases with high stemness scores had significantly increased expression of stem cell markers (SOX2, MYC, KRT18, BMI1, CD24) compared to those with low stemness scores (Fig. 3L).

Using the stemness scores to evaluate the relationship between stemness and autophagy confirmed that the expression of autophagy-related genes was upregulated in the high stemness scores group (Fig. 3M). Additionally, ATAC-seq analysis of high stemness patients reveals that most peaks are located within 1 kb of the transcription start site, particularly in promoter regions (Fig. 3N). These regions also show the greatest overlap with other genomic features, highlighting their key role in regulating gene expression in high stemness patients (Fig. 3O). GO analysis of genes in these promoter regions identifies those linked to stem cell maintenance and proliferation, suggesting their role in regulating promoter activity to support stem cell functions and promote tumor growth (Fig. 3P). Likewise, genes associated with enriched KEGG function in autophagy, p53 pathway, and Ras signaling pathway via analyzing the ATAC-seq data related to promoter regions (Fig. 3Q). Applying the stemness scores to predict potential molecular regulatory mechanisms and functions, GSEA analysis revealed that in addition to the regulation of autophagy, high stemness score groups were enriched in E2F targets, p53 signaling pathway, mTORC1 signaling, and other tumor-related pathways (Fig. 3R). Correlation analysis between the E2F family and stemness scores showed significant correlations between several E2F family transcription factors and stemness scores (Fig. 3S). Thereinto, E2F1 was the only gene in the E2F family among the top 10% of autophagy-related scores which showed a significant correlation with the stemness score (Fig. 3T, *r* = 0.54). E2F1 expression was significantly increased in the high stemness score group (Fig. 3U). By analyzing the group with high stemness scores in LUAD, it was found that E2F1 was enriched in regulation of autophagy, cell cycle, and the p53 signaling pathway (Fig. 3V). Furthermore, GO functional enrichment analysis of 53 autophagy genes co-expressed with E2F1 in LUAD tissues with high stemness scores suggested that E2F1 was involved in protein transport, protein localization, protein kinase activity, and protein phosphorylation in these groups (Fig. 3W).

To verify the unique biological advantage and clinical predictive value of E2F1, we compared it with classic stemness genes such as MYC and SOX2. E2F1 expression was significantly increased by the greatest multiple in the high stemness score group compared to SOX2 and MYC (Fig.S2A), as well as in the tumor and adjacent normal tissues group (Fig.S2B), early(I + II) and advanced stage (III + IV) group (Fig.S2C). Further analysis revealed that E2F1 enriched different functional pathways compared to MYC and SOX2. For instance, E2F1 mainly mediates the P53 signaling pathway and autophagy, while MYC enriches in RNA degradation, nod like receptor signaling pathway and T cell receptor signaling pathway, and SOX2 is involved in the ALPHA linolenic acid metabolism and Hedgehog signaling pathway (Fig. S2D-G). Regarding prognosis, E2F1 expression was associated with age, gender, smoking, stage, and tumor size, whereas MYC correlated with smoking, tumor size, and metastasis, and SOX2 was only associated with lymph node metastasis (Fig. S2H-J). Both E2F1 and MYC expression were positively correlated with poor prognosis. Among them, E2F1 exhibiting the most significant difference (*P* < 0.001), while SOX2 showed a negative correlation (Fig. S2K-M). Unlike the SOX2 and MYC, E2F1 also served as an independent prognostic factor (Fig. S2N). By analyzing the ROC curves for time-dependent survival predictions of 3/5/10 years, the high expression of E2F1 and MYC provided valuable prognostic information. E2F1 demonstrated better performance in prognosis compared to MYC (Fig. S2O-Q). Finally, the patients were divided into four sub-groups based on the “E2F1 expression/stemness level”. We found that, quite interestingly, the prognosis of double low-expression group was better, while the double high-expression group had the worst prognosis (Fig. S2R). However, no such effect was observed for SOX2 and MYC (Fig. S2S-T), confirming the unique role of E2F1 and stemness in survival.

### E2F1 promotes self-renewal and drug resistance in A549 spheroid cells but has no effect on H1299 spheroid cells

**Fig. 4:**
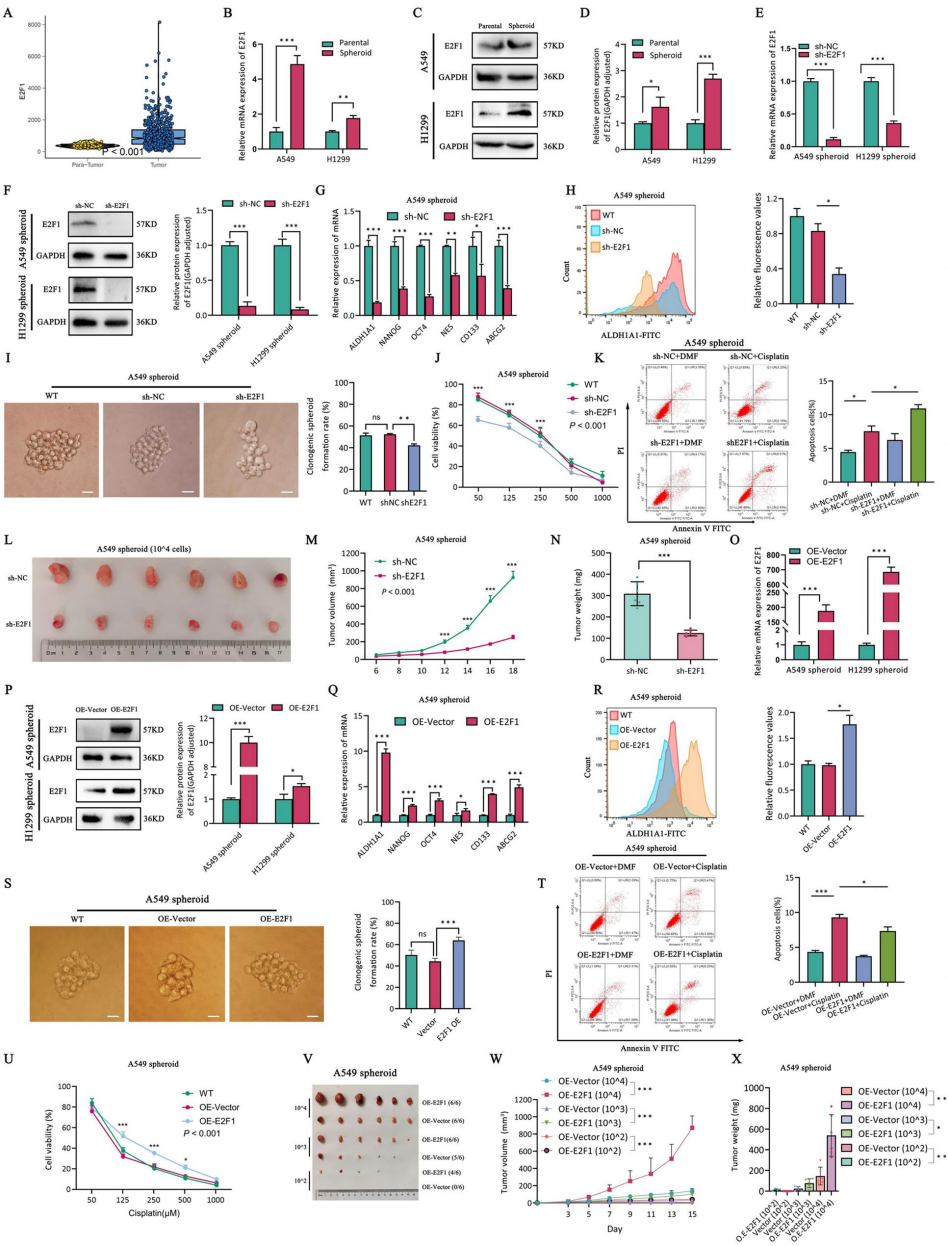
E2F1 promotes self-renewal and drug resistance of lung cancer stem cells. (**A**) E2F1 expression in LUAD patient. **B-D**. E2F1 expression in LUAD parental and spheroid cells. (**B**) mRNA expression. **C-D**. Protein expression and quantification assay. **E-F**. The expression of E2F1 in sh-NC and sh-E2F1 of LUAD spheroid cells. **E**. RT-qPCR. **F**. Western blotting and quantification assay. **G-I**. The self-renewal capability of A549 spheroid sh-NC and sh-E2F1 cells. **G**. mRNA abundance of CSCs marker. **H**. Flow cytometry analysis of ALDH1A1 expression in LUAD parental and spheroid cells. The Right panel represents quantification of ALDH1A1 expression. **I**. Single clone assay and quantification assay. **J-K**. The drug resistance of A549 spheroid sh-NC and sh-E2F1 cells. **J**. CCK8. **K**. The cell apoptosis by flow cytometry analysis and quantification assay. **L-N**. Tumor formation in nude mice following injection of A549 spheroid cells sh-NC and sh-E2F1, respectively. **L**. The image of tumor. **M**. Tumor growth curves. **N**. Tumor weight. **O-P**. The expression of E2F1 in OE-Vector and OE-E2F1 of LUAD spheroid cells. **O**. RT-qPCR. **P**. Western blotting and quantification assay. **Q-S**. The self-renewal capability of A549 spheroid OE-Vector and OE-E2F1 cells. **Q**. mRNA expression of stemness genes. **R**. ALDH1A1 expression by flow cytometry and quantification assay. **S**. Single clone assay and quantification assay. **T-U**. The drug tolerance of A549 spheroid OE-Vector and OE-E2F1 cells. **T**. Flow cytometry analysis and quantification assay. **U**. CCK8. **V-X**. The gradient tumor formation assays in A549 spheroid OE-Vector and OE-E2F1 cells in BALB/c nude mice. **V**. Tumor volume. **W**. Tumor growth curve. **X**. Tumor weight. *P < 0.05, **P < 0.01, ***P < 0.001

Given the relationship between E2F1 and stemness as well as autophagy, we analyzed the expression and function of E2F1 in LUAD and its CSCs. Firstly, the bioinformatic analysis indicated high E2F1 expression in LUAD compared with the adjacent group, indicating that it may function as an oncogene (Fig. 4A). Subsequently, compared to A549 parental cells, both mRNA (Fig. 4B) and protein levels of E2F1 (Fig. 4C-D) were found to be upregulated in LCSCs. Furthermore, knockdown of E2F1 in A549 spheroid cells (Fig. 4E-F), then self-renewal (Fig. 4G-I), drug resistance (Fig. 4J-K) and tumorigenicity in vivo (Fig. 4L-N) of A549 spheroid cells was also inhibited, whereas no such changes were observed in H1299 spheroid cells (Fig.S3A-E). Following that, overexpression of E2F1 in LCSCs (Fig. 4O-P) resulted in an enhanced self-renewal (Fig. 4Q-S) and increased drug resistance (Fig. 4T-U) of A549 spheroid cells. Further, the overexpression of E2F1, its tumorigenicity in vivo also showed a gradient increase of A549 spheroid cells (Fig. 4V-X), but the phenotypes described above were not observed in H1299 spheroid cells (Fig.S3F-J). In summary, these results confirmed that E2F1 is upregulated in LCSCs and promotes the self-renewal and drug resistance of A549 spheroid cells, but not in H1299 spheroid cells. Moreover, the intrinsic mechanisms underlying such differential phenomenon require investigation.

### E2F1 induces autophagy to enhance self-renewal and drug resistance in A549 spheroid cells but has no effect on H1299 spheroid cells

**Fig. 5:**
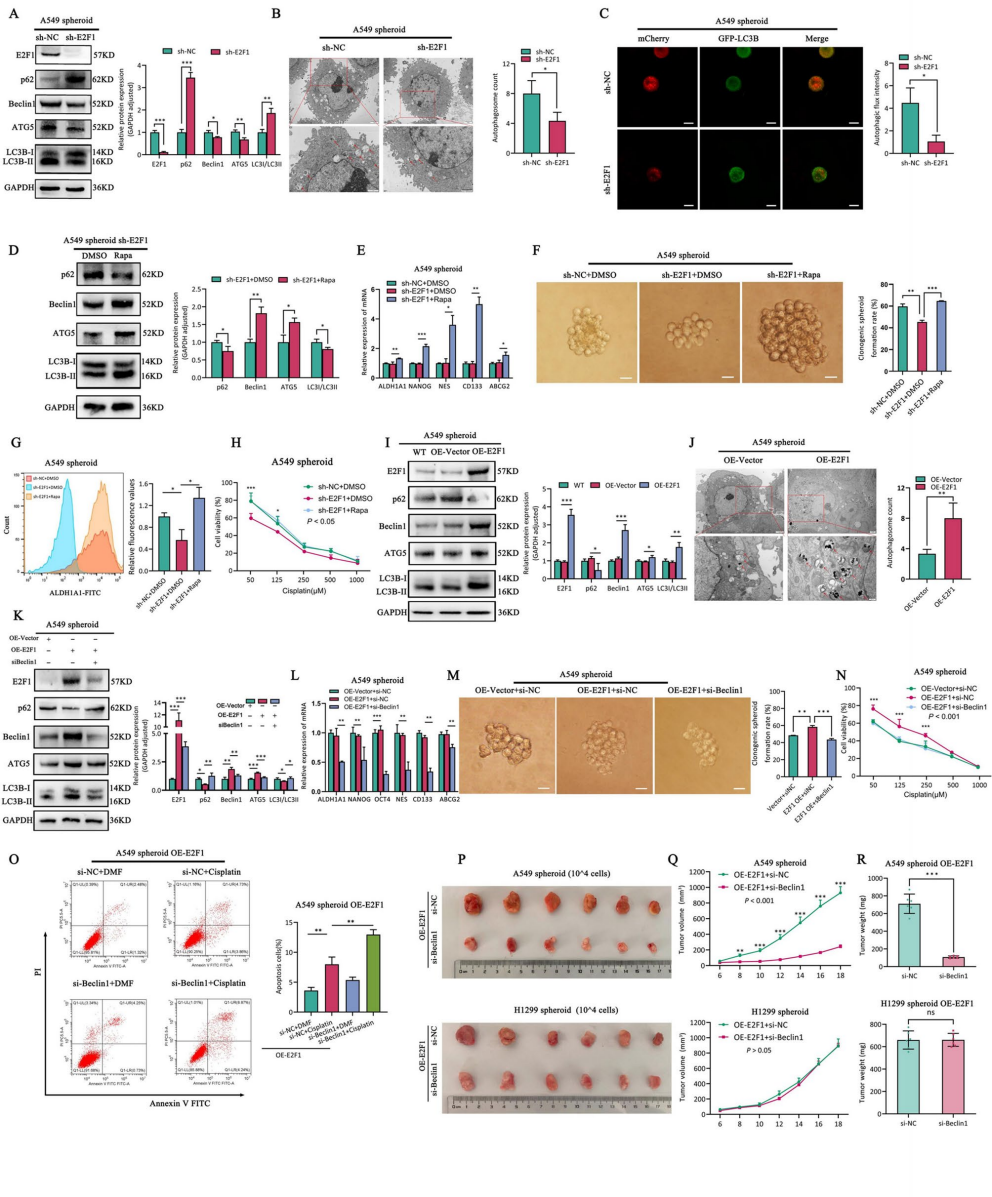
E2F1 enhances autophagy to promote self-renewal and drug resistance of lung cancer stem cells. **A-C**. The autophagy flux and quantification assay of A549 spheroid sh-NC and sh-E2F1 cells. (**A**) Western blotting. (**B**) TEM analysis. (**C**) Autophagic flow by confocal microscope. (**D**) Expression of autophagy relative protein and quantification assay in A549 spheroid sh-NC and sh-E2F1 cells incubated with Rapa by western blotting. **E-G**. The self-renewal capability of A549 spheroid sh-E2F1 cells incubated with Rapa and control. (**E**) RT-qPCR. (**F**) Single clone assay and quantification assay. (**G**) Flow cytometry analysis and quantification assay. (**H**) The proliferation activities of A549 spheroid sh-E2F1 cells by pre-incubated with Rapa and control, and treated with cisplatin. **I-J**. The autophagy flux and quantification assay of A549 spheroid OE-Vector and OE-E2F1 cells. (**I**) Western blotting. (**J**) TEM analysis. **K**. The autophagy protein expression and quantification assay of A549 spheroid OE-E2F1 + si-NC and OE-E2F1 + si-Beclin1 cells. **L-M**. The self-renewal ability of A549 spheroid OE-E2F1 + si-NC and OE-E2F1 + si-Beclin1 cells. **L**. RT-qPCR. **M**. Single clone assay and quantification assay. N-O. The drug tolerance of A549 spheroid OE-E2F1 + si-NC and OE-E2F1 + si-Beclin1 cells. **N**. CCK8. **O**. Flow cytometry assay and quantification assay. P-R. Subcutaneous tumors were harvested after LUAD spheroid OE-E2F1 + si-NC and OE-E2F1 + si-Beclin1 cells in BALB/c nude mice. **P**. Tumor volume. **Q**. Tumor growth curve. **R**. Tumor weight

To reveal whether E2F1 promotes the stemness and drug resistance of LCSCs via autophagy, we observed the decrease in autophagic activity in shE2F1-A549 spheroid cells (Fig. 5A-C), while no such changes were observed in shE2F1-H1299 spheroid cells (Fig.S4A-C). Subsequently, we activated autophagy in shE2F1-A549 spheroid cells by Rapamycin (Rapa) to reactivate autophagic activity (Fig. 5D), as well as the recovery of the self-renewal (Fig. 5E-G) and drug resistance (Fig. 5H). Similarly, we overexpressed E2F1 in LCSCs and observed an upregulation of autophagic activity in A549 spheroid cells (Fig. 5I-J), while there was no effect on H1299 spheroid cells (Fig.S4D-E). To confirm the regulation of self-renewal and drug resistance of LCSCs by the “E2F1-autophagy” axis, we upregulated E2F1 and then knocked down Beclin1 (Fig. 5K, Fig. S4F). The results showed that self-renewal (Fig. 5L-M) and drug resistance (Fig. 5N-O) were only affected in A549 spheroid cells, while there was no impact on H1299 spheroid cells (Fig.S4G-J). Meanwhile, similar results were found in in vivo experiment (Fig. 5P-R). Summarily, these results confirmed that E2F1 regulates autophagy to promote self-renewal and drug resistance in A549 spheroid cells, whereas no such findings were observed in H1299 spheroid cells.

### Autophagy regulation of ALDH1A1 enhances self-renewal and drug resistance in A549 spheroid cells

**Fig. 6:**
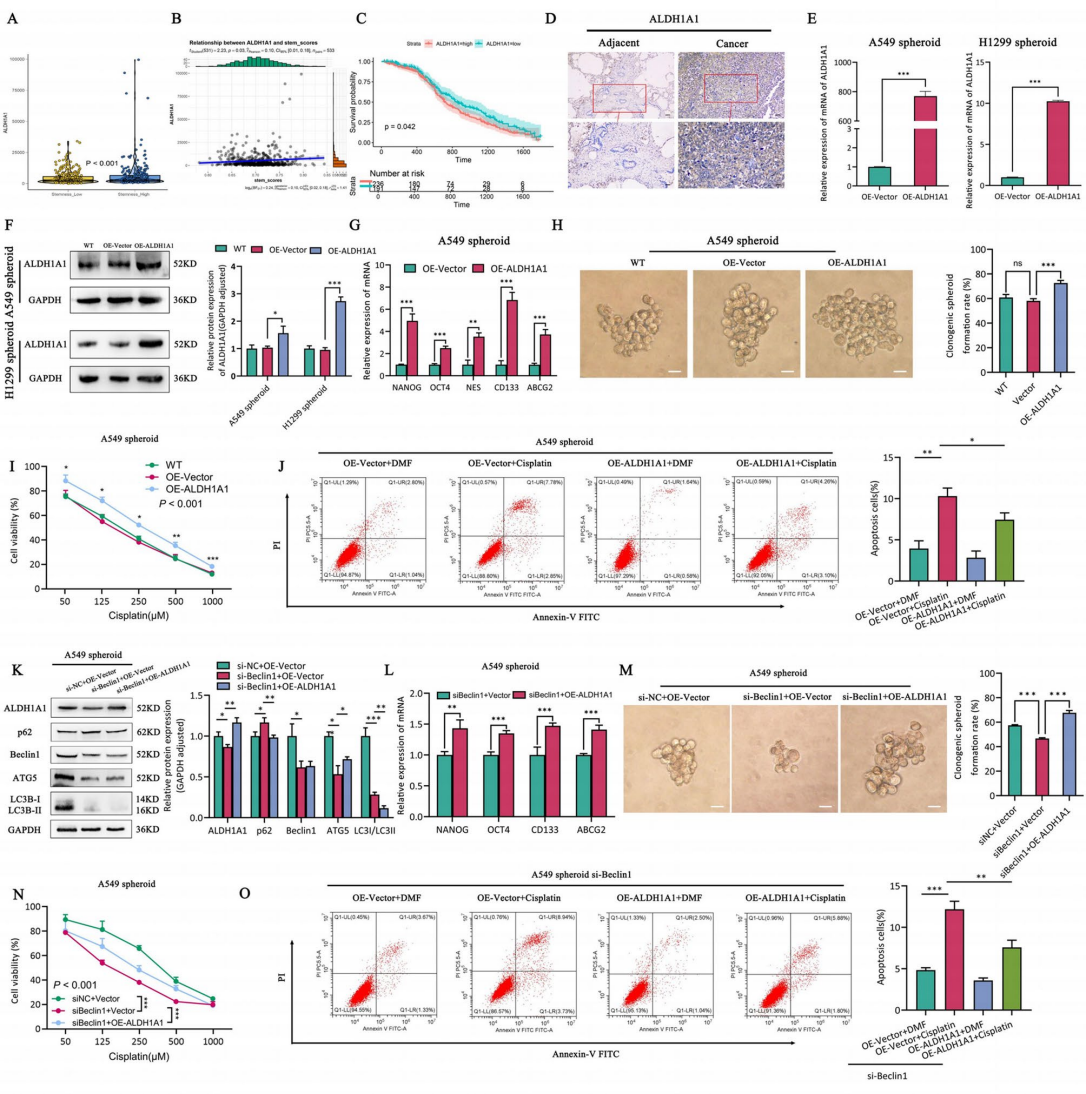
ALDH1A1 as stemness marker enhances self-renewal and drug resistance of lung cancer stem cells. (**A**) ALDH1A1 mRNA expression of stemness score high and low groups. (**B**) Correlation of ALDH1A21 expression and stemness in LUAD. (**C**) Kaplan-Meier estimates of the OS of patients with LUAD according to ALDH1A1 levels. (**D**) Immunohistochemical staining of ALDH1A1 in LUAD and its adjacent tissues, scale bar = 60 μm (upper panels), scale bar = 30 μm (lower panels). **E-F**. ALDH1A1 expression in LUAD spheroid OE-Vector and OE-ALDH1A1 cells. (**E**) mRNA expression. (**F**) Western blotting and quantification assay. **G-H**. The self-renewal activity of A549 spheroid OE-Vector and OE-ALDH1A1 cells. (**G**) CSCs markers by RT-qPCR. (**H**) Spheroid formation by single clone assay and quantification assay. **I-J**. The drug resistance of A549 spheroid OE-Vector and OE-ALDH1A1 cells. (**I**) The proliferation activity. (**J**) Flow cytometry assay and quantification assay. **K**. The autophagy relative protein expression and quantification assay of A549 spheroid si-Beclin1 + OE-Vector and si-Beclin1 + OE-ALDH1A1 cells. **L-M**. The self-renewal capability of A549 spheroid si-Beclin1 + OE-Vector and si-Beclin1 + OE-ALDH1A1 cells. **L**. RT-qPCR. **M**. Single clone assay and quantification assay. **N-O**. The therapy tolerance of A549 spheroid si-Beclin1 + OE-Vector and si-Beclin1 + OE-ALDH1A1 cells. **N**. CCK8 assay. **O**. Cell apoptosis by flow cytometry and quantification assay

Previous research has shown that ALDH1A1 is upregulated in various solid tumor CSCs [37], and ALDH1A1 conferred erlotinib resistance by inducing the ROS-RCS metabolic program in LUAD [35]. Regardingly, ALDH1A1 expression was found to be significantly upregulated in the high stemness group compared to the low stemness group as revealed through bioinformatics analysis, and positively correlated with cancer stemness (Fig. 6A-B). Moreover, survival analysis showed that high ALDH1A1 expression is associated with poor prognosis in LUAD (*P* = 0.042) (Fig. 6C). Furthermore, ALDH1A1 protein expression was upregulated in LUAD and positively correlated with tumor stage (*P* = 0.020) and distant metastasis (*P* = 0.006) (Fig. 6D; Table 1). In addition, we overexpressed ALDH1A1 in A549 spheroid cells and H1299 spheroid cells (Fig. 6E-F), and also observed a significant enhancement of self-renewal (Fig. 6G-H, Fig.S5A-B) and drug resistance (Fig. 6I-J, Fig.S5C-D) in LCSCs. The above results confirmed that ALDH1A1 is up-regulated in LCSCs and promoted its self-renewal and drug resistance.

**Table 1:** Relationships between the protein expression of ALDH1A1 and clinicopathological parameters in LUAD based on IHC detection

Variables	ALDH1A1 protein expression of LUAD			*P*-value
	Total (*n* = 95)	Low (*n* = 43)	High (*n* = 52)	
**Gender**				
Female	48	18	30	0.124
Male	47	25	22	
**Age**				
< 50	10	7	3	0.097
≥ 50	85	36	49	
**Stage**				
I + II	45	26	19	0.020*
III + IV	50	17	33	
**Tumor Invasion**				
T1 + T2	50	28	22	0.027
T3 + T4	45	15	30	
**Lymphatic metastasis**				
**-**	49	26	23	0.115
**+**	46	17	29	
**Distant metastasis**				
M0	45	27	18	0.006*
M1	50	16	34	

LUAD: lung adenocarcinoma. IHC: immunohistochemistry

* Bold values indicate *P* < 0.05

Subsequently, we inhibited autophagy by silencing Beclin1 and then activated ALDH1A1 (Fig. 6K, Fig.S5E-F). We observed a significant recovery of self-renewal capacity (Fig. 6L-M, Fig. S5G-H) and drug resistance (Fig. 6N-O, Fig. S5I-J) in both A549 and H1299 spheroid cells. In general, these results confirmed that ALDH1A1, acts as a downstream gene of “E2F1-autophagy-ALDH1A1” axis, and promoted self-renewal and drug resistance in LCSCs.

### E2F1-autophagy-ALDH1A1 axis enhances self-renewal and drug resistance in LCSCs in a p53-dependent manner

**Fig. 7:**
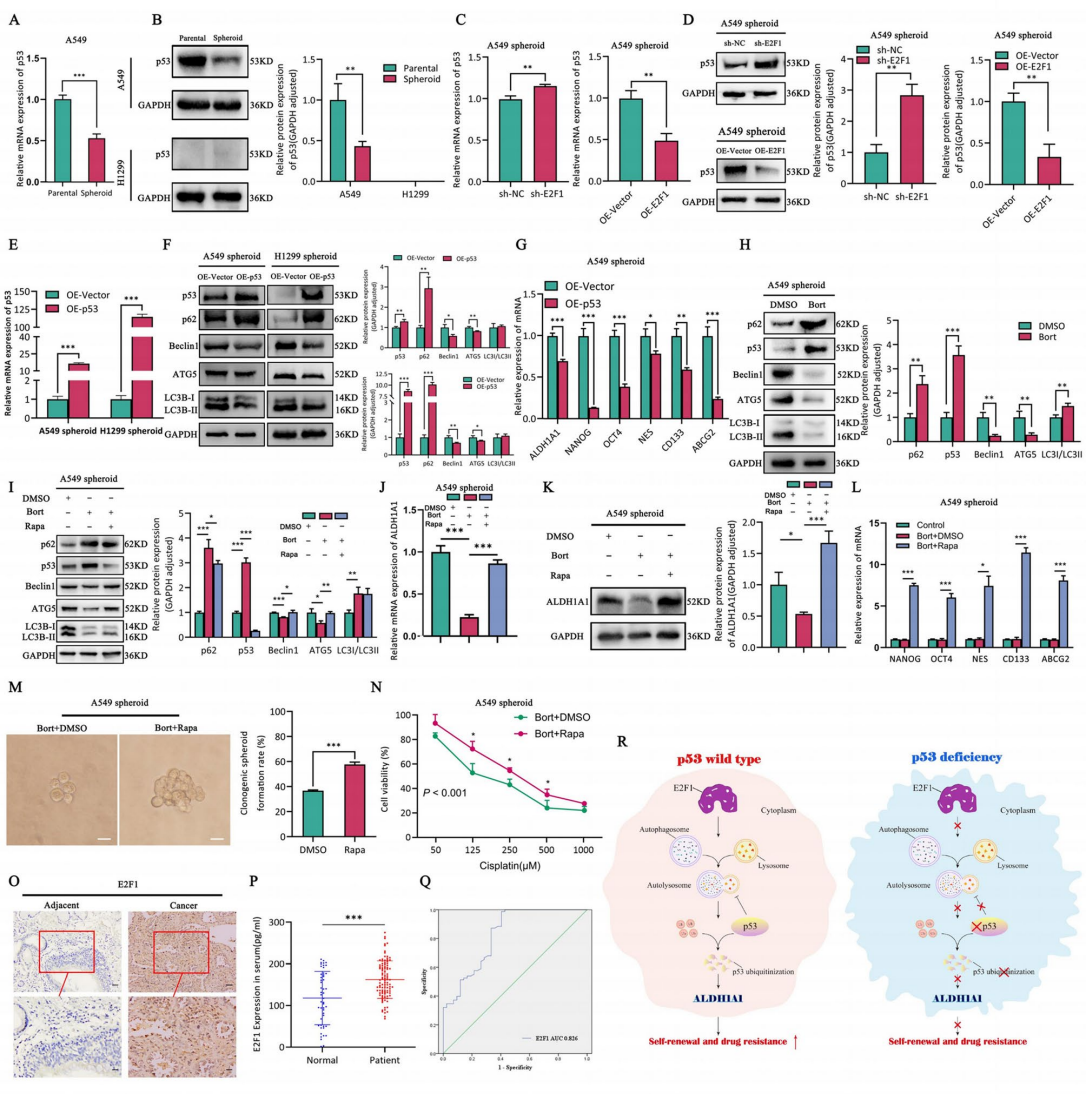
E2F1-autophagy-ALDH1A1 axis promotes self-renewal and drug resistance of lung cancer stem cells in a p53-dependent manner. **A-B**. P53 expression in LUAD parental and spheroid cells. (**A**) mRNA abundance. (**B**) Protein expression and quantification assay. (**C**) P53 mRNA expression in A549 spheroid sh-NC/sh-E2F1 and OE-Vector/OE-E2F1 cells. (**D**) P53 protein expression in A549 spheroid sh-NC/sh-E2F1 and OE-Vector/OE-E2F1 cells and quantification assay. (**E**) P53 mRNA expression in A549 spheroid/H1299 spheroid OE-Vector and OE-P53 cells. (**F**) Autophagy relative protein expression and quantification assay in LUAD spheroid OE-Vector and OE-p53 cells. (**G**) Stemness associated genes expression in A549 spheroid OE-Vector and OE-p53 cells. (**H**) Autophagy relative protein expression and quantification assay in A549 spheroid cells incubated with proteasome inhibitor Bortezomib. (**I**) Protein abundance and quantification assay of autophagy in A549 spheroid cells incubated with Bortezomib and Rapa. **J-K**. ALDH1A1 expression in A549 spheroid cells treated with Bortezomib and Rapa. (**J**) RT-qPCR. **K**. Western blotting and quantification assay. **L-M**. The self-renewal capability of A549 spheroid cells treated with Bortezomib and Rapa. **L**. mRNA abundance of stemness genes. **M**. Single clone assay and quantification assay. **N**. Cell viability of A549 spheroid cells treated with Bortezomib and Rapa by CCK8. **O**. Immunohistochemical staining of E2F1 in LUAD and its adjacent tissues, scale bar = 60 μm (upper panels), scale bar = 30 μm (lower panels). **P-Q**. The diagnostic assessment of E2F1 expression in LUAD patients and healthy groups. **P**. The protein abundance in serum. **Q**. ROC curve. **R**. The chart summary of “E2F1-autophagy-ALDH1A1” axis enhances self-renewal and drug resistance of lung CSCs in a p53 dependent manner

We have indicated that E2F1 differentially regulates self-renewal and drug resistance in A549 and H1299 spheroid cells. According to bioinformatic analysis (Fig. 3V), we validated that p53 expression was significantly reduced in A549 spheroid cells compared to A549 parental cells (Fig. 7A, B upper); whereas H1299 parental cells and H1299 spheroid cells exhibited p53 deletion (Fig. 7B lower). Subsequently, after knocking down and overexpressing E2F1, we found that the changes in p53 protein levels and p53 mRNA expression were negatively correlated with E2F1 (Fig. 7C-D). Since p53 has a dual regulatory role in autophagy, we overexpressed p53 in A549 and H1299 spheroid cells (Fig. 7E), and the expression of autophagy-related proteins expression was significantly reduced (Fig. 7F), as well as a notable downregulation of stemness gene mRNA levels, especially ALDH1A1 in A549 spheroid cells (Fig. 7G). Next, we used the proteasome inhibitor Bortezomib (Bort) to inhibit p53 ubiquitination and noticed significant decrease in autophagy protein expression in A549 spheroid cells (Fig. 7H). Then, we incubated A549 spheroids with combination of Bort and Rapa, and found that the expression of autophagy-related proteins, ALDH1A1 mRNA and protein in A549 spheroid cells was rescued (Fig. 7I-K), and their self-renewal (Fig. 7L-M) and drug resistance (Fig. 7N) were also significantly restored. To sum up, E2F1 enhances autophagy, also strengthens the ubiquitination and degradation of p53, thereby enhancing ALDH1A1 expression and ultimately promoting self-renewal and drug resistance in A549 spheroid cells.

We also investigated whether the differences observed in A549 and H1299 spheroid cells, apart from mainly attributed to p53 status, are related to KRAS/BRAF signaling or oxidative metabolism. Initially, we knocked down KRAS and found that stemness (Fig. S6A), autophagy (Fig. S6B-C), and drug resistance (Fig. S6D-F) in A549 and H1299 spheroid cells were significantly affected. Similarly, treatment with the KRAS/BRAF pathway inhibitor Braftide in A549 and H1299 spheroid cells yielded similar results (Fig. S6G-L). Furthermore, knocking down the key oxidative metabolism gene HIF1A significantly decreased the oxygen consumption rate (OCR) in both A549 and H1299 spheroid cells (Fig. S7A), and similarly impacted stemness (Fig. S7B), autophagy (Fig. S6C-D), and drug resistance (Fig. S7E-F). These findings suggest that KRAS and oxidative metabolism are not responsible for the observed differences in the autophagy-stemness axis in A549 and H1299 spheroid cells.

Furthermore, to evaluate the clinical value of E2F1 as a biomarker for LUAD, we analyzed the expression of E2F1 in serum and tissue samples from LUAD patients. We observed that E2F1 protein expression was significantly increased in LUAD tissues compared to the adjacent tissues (Fig. 7O), and was positively correlated with tumor stage (*P* = 0.032), invasion (*P* = 0.049) and distant metastasis (*P* = 0.032) (Table 2). Additionally, to be used as a novel no-invasion biomarker, E2F1 expression was upregulated in the serum of LUAD patients compared to the healthy group (Fig. 7P), and the area under the ROC curve (AUC) was 0.90 (Fig. 7Q). Meanwhile, the serum levels of E2F1 also positively correlated with tumor stage (*P* = 0.030) and distant metastasis (*P* = 0.020). These results clearly indicated that E2F1 confers significant diagnostic value in LUAD. Comprehensively, the results suggested that p53 inhibits autophagy in LCSCs, revealing the underlying mechanism by which the “E2F1-autophagy-ALDH1A1” axis mediates autophagy induced self-renewal and drug resistance in a p53-dependent manner (Fig. 7R), confirming E2F1 as a promising biomarker for LUAD (Tables 2 and 3).

**Table 2:** Relationships between the protein expression of E2F1 and clinicopathological parameters in LUAD based on IHC detection

Variables	E2F1 protein expression of LUAD			*P*-value
	Total (*n* = 95)	Low (*n* = 46)	High (*n* = 49)	
**Gender**				
Female	48	26	22	0.257
Male	47	20	27	
**Age**				
< 50	10	6	4	0.439
≥ 50	85	40	45	
**Stage**				
I + II	45	27	18	0.032*
III + IV	50	19	31	
**Tumor Invasion**				
T1 + T2	50	29	21	0.049*
T3 + T4	45	17	28	
**Lymphatic metastasis**				
**-**	49	28	21	0.079
**+**	46	18	28	
**Distant metastasis**				
M0	45	27	18	0.032*
M1	50	19	31	

LUAD: lung adenocarcinoma. IHC: immunohistochemistry

* Bold values indicate *P* < 0.05

**Table 3:** Relationships between the expression of E2F1 and clinicopathological parameters in LUAD based on ELISA

Variables	E2F1 mRNA expression of LUAD			*P*-value
	Total (*n* = 100)	Low (*n* = 56)	High (*n* = 44)	
**Gender**				
Female	49	27	22	0.859
Male	51	29	22	
**Age**				
< 50	7	3	4	0.468
≥ 50	93	53	40	
**Stage**				
I + II	44	30	14	0.030*
III + IV	56	26	30	
**Tumor Invasion**				
T1 + T2	53	32	21	0.349
T3 + T4	47	24	23	
**Lymphatic metastasis**				
-	44	28	16	0.173
+	56	28	28	
**Distant metastasis**				
M0	54	36	18	0.020*
M1	46	20	26	

LUAD: lung adenocarcinoma. ELISA: enzyme linked immunosorbent assay

* Bold values indicate *P* < 0.05

## Discussion

CSCs are the core component of tumors, possessing high potential for self-renewal and drug resistance, which are the primary elements inducing oncogenesis, progression and poor prognosis. Due to the intricate mechanisms of stemness maintenance of CSCs, much of the function and biology of autophagy remains elusive. In this study, we determined the expression and functions of E2F1 and autophagy in LCSCs, revealing the underlying mechanism of the “E2F1-autophagy-ALDH1A1” axis in enhancing self-renewal and drug resistance of LCSCs through a p53 dependent mechanism, followed by assessing the diagnostic efficacy of E2F1 as a biomarker for LUAD.

Firstly, we demonstrated that LCSCs exhibit higher autophagy activity compared to LUAD parental cells (Fig. 1J-N). Furthermore, autophagy has been shown to promote self-renewal and drug resistance in A549 spheroid cells (Fig. 2). Prior to this study, there has been limited perception about the expression and function of autophagy in LCSCs, with few studies suggesting that mitophagy could promote the stemness of LCSCs [38]. Concurrently, it is well-established that autophagy exerts dualistic effect on CSCs across various solid tumors. This complexity arises from diverse molecular mechanisms influenced by tumor type, specific genetic drivers, disease stage, and critically, the dynamic tumor microenvironment (TME) conditions. Notably, our observation that autophagy supports LCSC properties aligns with its well-documented pro-survival and pro-stemness functions in CSCs under specific stresses. For instance, under common TME stresses such as hypoxia or nutrient deprivation, autophagy is frequently upregulated and serves as a crucial cytoprotective mechanism [39]. Under these conditions, autophagy clears damaged organelles and recycles macromolecules, thereby maintaining metabolic homeostasis, reducing oxidative stress, and preventing lethal damage, which collectively enhances CSC survival and preserves their stem-like state [28, 40]. This adaptive role underscores how autophagy can fortify CSC properties under adverse conditions to drive tumor progression and therapy resistance. The molecular pathways underpinning autophagy’s impact on stemness also exhibit significant heterogeneity. In head and neck squamous cell carcinoma (HNSCC) CSCs, autophagy promotes stemness specifically through a non-canonical FOXO3/SOX2 signaling axis [41], while in pancreatic cancer stem cells, it contributes predominantly to gemcitabine resistance [42]. Contrarily, some studies have also indicated that excessive autophagy can lead to the loss of tumor stem cell function and even cell death. In pancreatic ductal adenocarcinoma (PDAC) CSCs, increased autophagic activity under hypoxic and nutrient-deprived conditions makes them more prone to necroptosis, reducing self-renewal and stemness gene expression [43]. In glioma-initiating cells (GICs), autophagy suppresses stemness and tumorigenicity by inhibiting the activation of the pro-stemness Notch1 signaling pathway [44]. This diametrically opposite effect in GICs emphasizes that autophagy’s ultimate functional output in CSCs is not universal in different tumor type. Within this complex autophagy functions in CSCs, our study defined a pro-stemness and pro-resistance role for autophagy in LCSCs derived from the A549 model. We not only demonstrate elevated basal autophagy in LCSCs but also establish its essential role in maintaining key CSC properties, supporting the idea that autophagy critically strengthens CSC traits, thereby promoting tumor progression and revealing a potential therapeutic vulnerability in LUAD. The different functional outputs of autophagy in different tumors may be finely regulated by tumor-intrinsic drivers (such as mutational background, p53 status) and external stressors (such as hypoxia, nutrient deprivation). These variables represent key directions for future research.

Secondly, we revealed a highly expressed E2F1 in LCSCs that enhanced its self-renewal and drug resistance by augmenting autophagy (Figs. 3, 4 and 5). According to previous research, Fouad S [45] demonstrated E2F1 as a transcription factor for tumor advanced development. In gastric cancer stem cells, E2F1 acts as a downstream transcription factor of circFAM73A/miR-490-3p/HMGA2 axis, contributing to maintain its stemness [17]. Here, we observed the changes in self-renewal, drug resistance and oncogenicity of LCSCs through E2F1 regulation (Fig. 4). These results reported that E2F1 strengthened self-renewal and drug resistance in A549 spheroid cells, also enriched the knowledge of expression and function of E2F1 in LCSCs, and E2F1 as an oncogene which enhanced self-renewal and tumorigenicity of CSCs. Moreover, we innovatively proposed that E2F1 encourages self-renewal and drug resistance of A549 spheroid cells by enhancing autophagy (Fig. 5). Based on the exploration of CSCs stemness and other characteristics, we revealed E2F1 promotes autophagy in A549 spheroid cells (Fig. 5A-D, I-K) and enhances their self-renewal capacity (Fig. 5E-G, L-M), drug resistance (Fig. 5H, N-O), and oncogenicity in vivo (Fig. 5P-R upper). Furthermore, bioinformatics further uncovered that E2F1 exhibited unique biological advantage in terms of promoting oncogenesis and progression, as well as clinical prognostic value via comparing with classic stemness genes such as MYC and SOX2 (Fig.S2). Hence, the present study underscores the crucial role of E2F1 in enhancing the self-renewal, drug resistance, and oncogenic potential of LCSCs by promoting autophagy.

Thirdly, the notable finding is that high expression of E2F1 enhances autophagy leading to ubiquitination and degradation of p53, which in turn promotes self-renewal and drug resistance of LCSCs. Moreover, the bidirectional role of p53 in autophagy regulation has been extensively documented, with p53 exhibiting both pro- and anti-autophagic functions depending on its subcellular localization and cellular context. Nuclear p53 has been shown to induce autophagy through transcriptional activation of pro-autophagic genes such as DRAM, DAPK-1, and PINK1. For instance, nuclear p53 can activate the PINK1 gene, a key regulator of mitochondrial autophagy, thereby promoting autophagic activity [46, 47]. Conversely, cytoplasmic p53 can inhibit autophagy through non-transcriptional mechanisms. It has been reported that cytoplasmic p53 suppresses autophagy by interacting with Beclin-1, leading to its degradation via the ubiquitin-specific peptidases USP10 and USP13, and by inhibiting the AMPK/mTOR signaling pathway [31, 48]. These findings highlight the complex and context-dependent regulation of autophagy by p53, especially in different subcellular localizations and cellular environments, where p53 can exert opposing functions. In addition, the dysfunction of p53 in tumor cells is often closely associated with the overactivation of MDM2. MDM2 overexpression enhances the ubiquitination of p53, thereby regulating its nuclear export [49–51]. This cascade of events governs the subcellular localization of p53 and exerts a potential regulatory influence on cellular autophagy. In the present study, E2F1-induced autophagy leads to the degradation of ubiquitinated p53, which may shift the balance towards a pro-autophagic phenotype, thereby enhancing LCSC stemness and drug resistance. This underscores the intricate interplay between E2F1, autophagy, and p53 in regulating LCSC properties. E2F1-induced autophagy selectively clears cytoplasmic p53 in A549 spheroid cells, relieving its inhibition on autophagy and creating a positive feedback loop that enhances autophagic activity (Fig. 7). While the reduction of nuclear p53 may weaken its transcriptional pro-autophagic function, the net effect of cytoplasmic p53 degradation significantly favors autophagy activation (Fig. 7I-N). Additionally, MDM2-mediated ubiquitination of nuclear p53 exposes its nuclear export signal (NES), allowing p53 to interact with CRM1 and be transported to the cytoplasm for ubiquitin-mediated degradation, which may enhance autophagic activity [52, 53]. This mechanism fails to operate in p53-deficient H1299 cells, confirming the central role of p53 (Fig. S4-5). By integrating the theory that subcellular localization determines p53 function with the regulatory role of E2F1, our study uncovers a novel mechanism for sustained autophagy activation in LCSCs. The E2F1-p53 axis essentially breaks the bidirectional balance of p53, pushing the cells into a pro-survival autophagic state, thereby creating a microenvironment in tumor cells that favors proliferation and resistance. Noteworthy, Morkel et al. reported that KRAS/BRAF signaling pathways influence CSC behavior and resistance in colorectal cancer [54]. Additionally, alterations in oxidative metabolism are crucial for regulating CSC properties, as they can switch between oxidative phosphorylation and glycolysis to maintain homeostasis and support tumor growth [55]. This is in line with our findings, where both KRAS and oxidative metabolism influence the autophagy-stemness axis in A549 and H1299 spheroid cells, modulating autophagic flux and regulating the stem cell-like characteristics of tumor cells (Fig. S6-7). Although KRAS and oxidative metabolism play a role in regulating stemness, autophagy and drug resistance, these factors do not explain the differences observed between A549 and H1299 spheroid cells. These findings strengthen the generality of our conclusion that p53 status is the primary determinant in regulating the “autophagy-stemness” axis.

According to the findings of the E2F1-autophagy-p53 regulatory axis in tumor stem cell resistance mechanisms in this study, we further explore the feasibility of a triple therapy involving E2F1 inhibitors, autophagy regulators, and MDM2-p53 axis inhibitors as a potential strategy to overcome tumor stem cell resistance. First, E2F1 promotes the self-renewal and drug resistance of LCSCs by regulating autophagy. Our research indicates that inhibiting E2F1 effectively disrupts the self-renewal feedback loop of LCSCs. Additionally, previous studies have identified E2F1 as a critical factor in regulating tumor stem cell characteristics and signaling pathways [56], playing a central role in chemotherapy resistance and mediating autophagy induced by gemcitabine in pancreatic cancer cells [57]. Polager et al. demonstrated that E2F1 directly regulates autophagy by transcriptionally upregulating autophagy-related genes such as LC3, ATG1, and DRAM [20]. Second, autophagy inhibitors (such as 3-MA) promote the accumulation of p53 in the cytoplasm, enhancing the apoptotic effects of tumor cells [31]. However, this mechanism alone may trigger p53 nuclear export, leading to the activation of alternative escape pathways. Finally, MDM2 inhibitors stabilize nuclear p53 and activate pro-apoptotic genes, further enhancing tumor cell apoptosis [58]. However, MDM2 monotherapy has therapeutic limitations. Combining E2F1 inhibitors, autophagy inhibitors, and MDM2 antagonists presents a potential multi-target therapeutic strategy. By inhibiting E2F1, this approach disrupts the self-renewal and autophagy processes of LCSCs, blocking the self-renewal loop at its origin. At the same time, autophagy inhibitors promote the accumulation of p53 in the cytoplasm, overcoming the p53 nuclear export defect caused by autophagy inhibition alone. MDM2 inhibitors stabilize nuclear p53, amplifying the apoptotic effects. The synergistic action of E2F1 inhibitors, autophagy regulators, and p53 MDM2 inhibitors, through the combined inhibition of E2F1-driven stemness/autophagy, blocking cytoplasmic p53 degradation, and preventing nuclear p53 inactivation, can effectively block the key escape pathways of LCSCs. This innovative strategy holds significant potential in overcoming the current treatment bottlenecks associated with LCSCs, providing a robust theoretical foundation for the development of more effective precision therapies.

The fourth innovative observation in our study is that ADLH1A1 acts downstream of “E2F1-autophagy” axis, to enhance the self-renewal and drug resistance of LCSCs. Previous reports on ALDH1A1, as a marker of cell stemness, have been verified in various solid tumors to promote self-renewal, drug resistance, and tumor development [59, 60], which is consistent with the conclusions of this study (Fig. 6A-H, Fig.S5A-E). Following this, we showed the upregulation of ALDH1A1, and self-renewal and drug resistance of A549 spheroid cells by p53 ubiquitination and activation of autophagy (Fig. 7I-N). This molecular mechanism discovery holds potential clinical application value. Previous studies have reported the potential of serum ALDH1A1 as a non-invasive biomarker for non-small cell lung cancer (NSCLC). Combined with the present findings that ALDH1A1 is directly regulated by the E2F1-autophagy axis, this creates a strong translational narrative. Our clinical data further validate this, showing that ALDH1A1 expression is upregulated in NSCLC tissues and significantly correlates with tumor prognosis, progression, and distant metastasis (Fig. 6C-D; Table 1). This pattern is similar to the clinical profile of its upstream regulator E2F1 (Fig. 7O; Table 2), indicating that detection of E2F1 expression in serum (AUC = 0.826, Fig. 7Q) could serve as a non-invasive biomarker to identify LUAD driven by this pathway. The dependency of LCSC self-renewal and drug resistance on ALDH1A1 highlights its significant therapeutic potential. Inhibiting ALDH1A1 or its upstream activators E2F1/autophagy may represent a promising therapeutic approach to eradicate therapy-resistant LCSCs.

Finally, it was also confirmed using clinical samples that E2F1 levels were markedly high in LUAD and positively correlated with clinical parameters such as tumor stage, invasion, and distant metastasis (Table S2-3). Importantly, we demonstrate that high E2F1 expression is mechanistically linked to increased tumor stemness, a relationship validated not only in clinical cohorts (Figs. 3S-U and 4A, S2K, S2N) but also functionally supported by our in vivo nude mouse tumor formation assays, where tumors driven by E2F1 exhibited enhanced tumorigenicity, mirroring the stemness phenotype observed in patients. This direct experimental bridge between clinical observations and in vivo mechanisms underscores the biological relevance of the E2F1-stemness axis. Survival analysis of the subgroup with E2F1 expression and stemness level further solidified this connection, namely the patients with concurrent high E2F1 expression and stemness scores suffered the worst outcomes (Fig. S2R-T). Moreover, ELISA confirmed E2F1 as a highly valuable clinical biomarker (AUC = 0.90; sensitivity 82.00%, specificity 80.30%; Fig. 7P-Q). Few literatures have investigated that E2F1 promoted stem cell characteristics and drove malignant progression in glioblastoma through transcriptional regulation [61], which has also been associated with tumor stemness, chemotherapy resistance, and patient prognosis by regulating autophagy through LC3, ATG1, and DRAM [20, 57]. However, the clinical translational value of the E2F1-stemness-prognosis relationship in LCSCs remains unclear. This study provides new insights by confirming the causal relationship between high E2F1 expression and enhanced tumor stemness through both mechanistic experiments and clinical validation. This association was functionally supported by xenograft tumorigenesis experiments in nude mice (Fig. 4L-O), where E2F1-driven tumors exhibited stronger tumorigenicity, enhanced stemness, and tumor growth-promoting characteristics. These in vivo findings were consistent with the clinical stemness and invasiveness phenotypes, as validated by clinical cohort data (Figs. 3R-U and 4A, S2K, S2N), suggesting that the pathophysiological mechanisms observed in animal models align with patient outcomes. ELISA results further established E2F1 as a high-value clinical biomarker (Fig. 7P-Q). Clinical data reveal a significant correlation between E2F1 expression and stemness marker expression (Fig. 3T), as well as its role in promoting tumor metastasis progression (Table 2). High E2F1 expression is positively correlated with poor prognosis (Figure S2K). Additionally, clinical data confirm that patients with high stemness phenotypes have worse outcomes (Fig. 3J-K). Survival analysis of the subgroup with both high E2F1 expression and high stemness scores further reinforced this association, with these patients experiencing the worst prognosis (Fig. S2R-T). This study not only uncovers the underlying mechanisms but also establishes a clinical correlation, focusing primarily on the link between E2F1 expression levels and prognosis. Based on above, we propose the development of an E2F1-stemness-prognosis prediction model using patient-derived models, such as Patient-Derived Xenograft (PDX), which will deepen our understanding of the causal relationship between this pathway and clinical outcomes, thus advancing the translation of precision medicine.

In conclusion, this study revealed a novel mechanism of E2F1 function in enhancing autophagy to upregulate ALDH1A1 expression, and promoting self-renewal and drug resistance of LCSCs in a p53-dependent manner. Moreover, we also proposed E2F1 as a potential biomarker and therapeutic target for patients with LUAD. Our study provided new insights into the regulatory mechanism of CSCs, their characteristics, and functional implications in the investigation of the oncogenicity and advanced.

## Supplementary Information

Below is the link to the electronic supplementary material.

Supplementary Material 1

## Data Availability

The datasets presented in this study can be found in online repositories or can be accessed upon request from the corresponding authors.
