# Supplementary material for: E2F1-autophagy-ALDH1A1 axis enhances self-renewal and drug resistance of lung cancer stem-like cells in a p53-dependent manner
Source: J Exp Clin Cancer Res. 2025 Aug 30;44:261. doi: 10.1186/s13046-025-03506-4 (PMC12398038; doi:10.1186/s13046-025-03506-4)
Supplement: Supplementary file 1 — Supplementary Material 1 [file 13046_2025_3506_MOESM1_ESM.docx]

**Supplementary Materials and methods**

**1. Clinical characteristics and differential analysis**

LUAD samples were divided into two groups according to mRNA expressions of E2F1, SOX2 and MYC of the two groups were evaluated based on R analysis. Clinical data such as gender, age, smoking, and TNM stages were also downloaded from TCGA data portal. The original data from TCGA was normalized and analyzed by R language.

**2. OCR assay**

The oxygen consumption rate (OCR) fluorescence test box (Elabscience, China, E-BC-F068) was used to determine the extracellular acidification rate and oxygen consumption rate according to the manufacturer's instructions. The cells were inoculated into 96-well plates at a density of 2 × 10^5^/ml. According to the experimental design, the corresponding drugs were added to stimulate the cells. After incubation at 37 °C in dark for 30 min, 100 μl working solution was added to each well. Fluorescence microplate reader was used to detect the fluorescence value of each well at the excitation wavelength of 490 nm and the emission wavelength of 535 nm. The OCR rate was calculated according to the time curve of fluorescence value.

**3. Supplement antibody**

The primary antibodies: KRAS Polyclonal antibody (Proteintech, 12063-1-AP), MEK1/2 (Proteintech, 11049-1-AP), Phospho-MEK1/MEK2 (Abclonal, AP1349) and HIF1a (Abmart, P50517R1).

**Supplementary Figure**


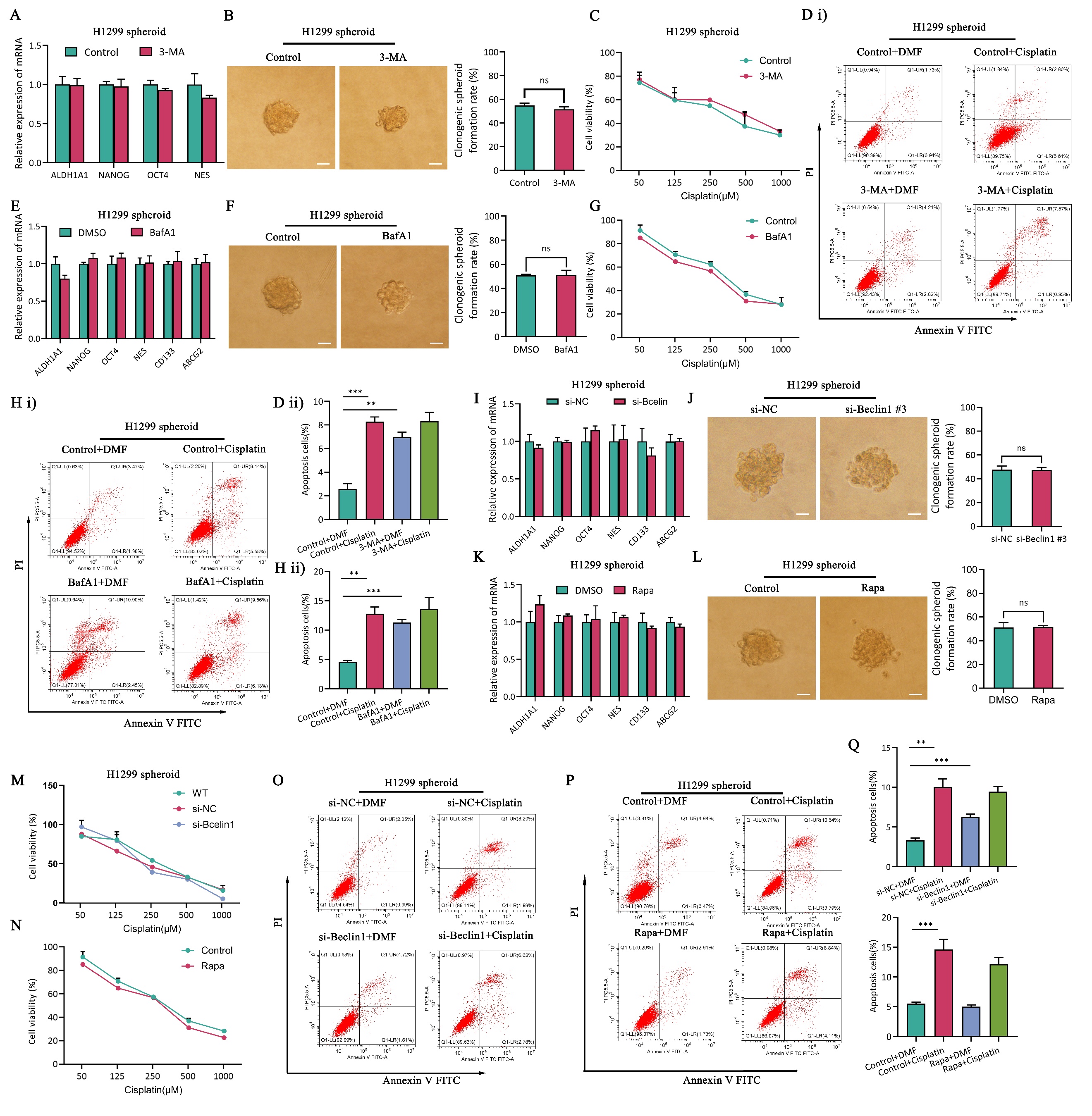


A. Stemness associated genes expression in H1299 spheroid cells treated with 3-MA and negative control. B. Spheroid formation by single clone assay in H1299 spheroid cells treated with 3-MA and negative control. C-D. The drug resistance of H1299 spheroid cells treated with 3-MA and negative control. C. CCK8 assay. D. Apoptosis analysis by flow cytometry. E. Stemness associated genes expression in H1299 spheroid cells treated with BafA1 and negative control. F. Spheroid formation by single clone assay in H1299 spheroid cells treated with BafA1 and negative control. G-H. The drug resistance of H1299 spheroid cells treated with BafA1 and negative control. G. CCK8 assay. H. Apoptosis analysis by flow cytometry. I. Stemness associated genes expression in H1299 spheroid si-Beclin1 and si-NC cells. J. Spheroid formation by single clone assay in H1299 spheroid si-Beclin1 and si-NC cells. K. Stemness associated genes expression in H1299 spheroid cells treated with Rapa and negative control. L. Spheroid formation by single clone assay in H1299 spheroid cells treated with Rapa and negative control. M-N. The drug resistance assay by CCK8. M. H1299 spheroid H1299 spheroid si-Beclin1 and si-NC cells. N. H1299 spheroid cells treated with Rapa and negative control. O-Q. Apoptosis analysis by flow cytometry. **P* < 0.05, ***P* < 0.01, ****P* < 0.001.


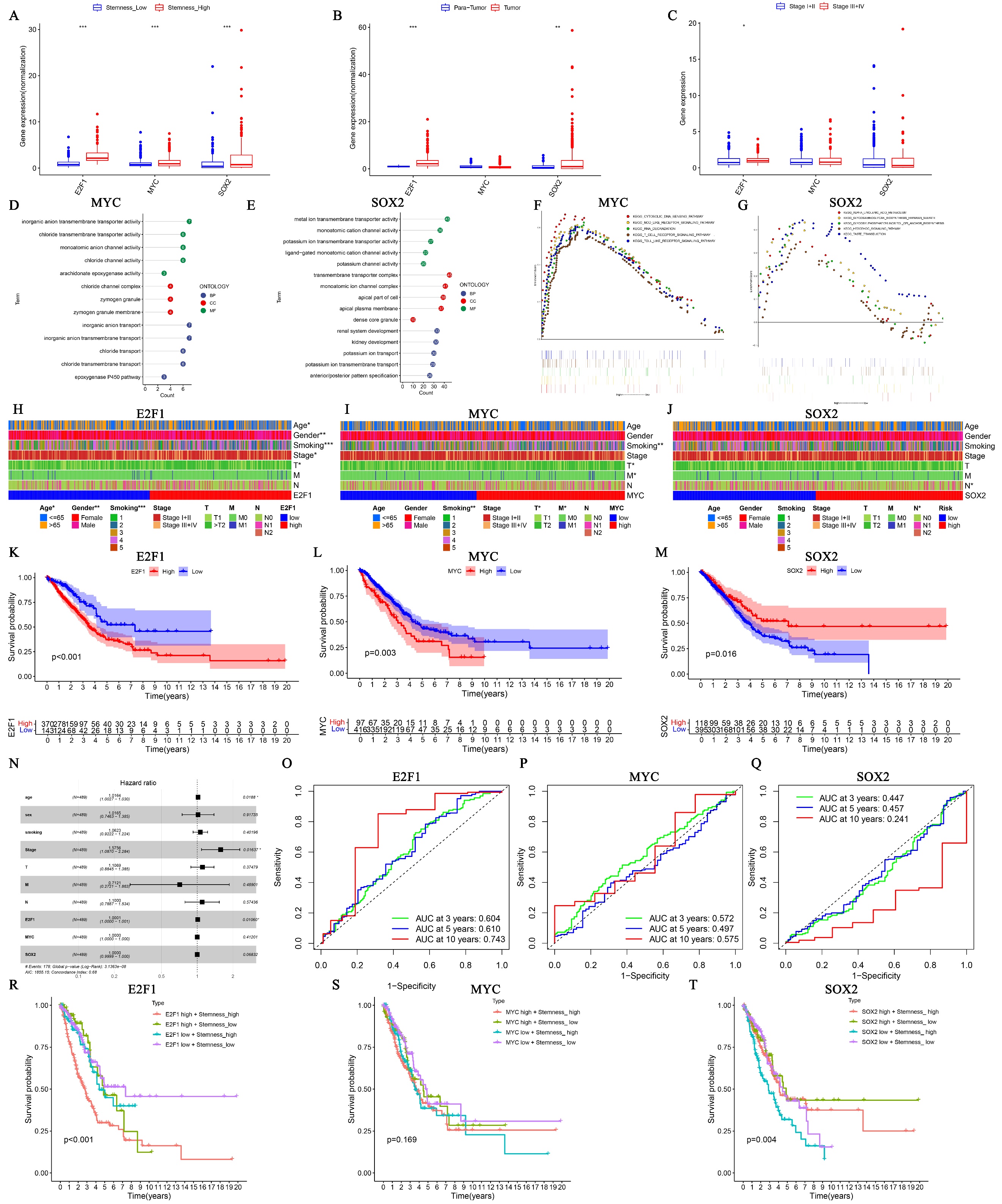


**FigureS2.** **The biological function and clinical predictive value of E2F1, MYC, and SOX2 in LUAD.**

A. The mRNA expression of E2F1, MYC and SOX2 of stemness score high and low groups in LUAD. B. The mRNA expression of E2F1, MYC and SOX2 of Para-tumor and tumor tissues in LUAD. C. The mRNA expression of E2F1, MYC and SOX2 of early and advanced stage in LUAD. D-E. GO enriched analysis of MYC and SOX2 in LUAD patients. F-G. KEGG enrichment analysis of MYC and SOX2 in LUAD patients. H-J. Heatmap showing the correlation of E2F1, MYC and SOX2 gene expression with clinical indicators in LUAD patients. K-M. Survival curves for LUAD patients based on the expression of E2F1, MYC and SOX2. N. Multivariate analysis of the correlation of E2F1, MYC and SOX2 gene expression with OS among LUAD. O-Q. ROC curve for evaluating the predictive value of E2F1, MYC and SOX2 in prognosis of LUAD patients. R-T. Kaplan-Meier survival curves illustrate the survival probabilities of sub-groups based on gene expression and stemness scores.


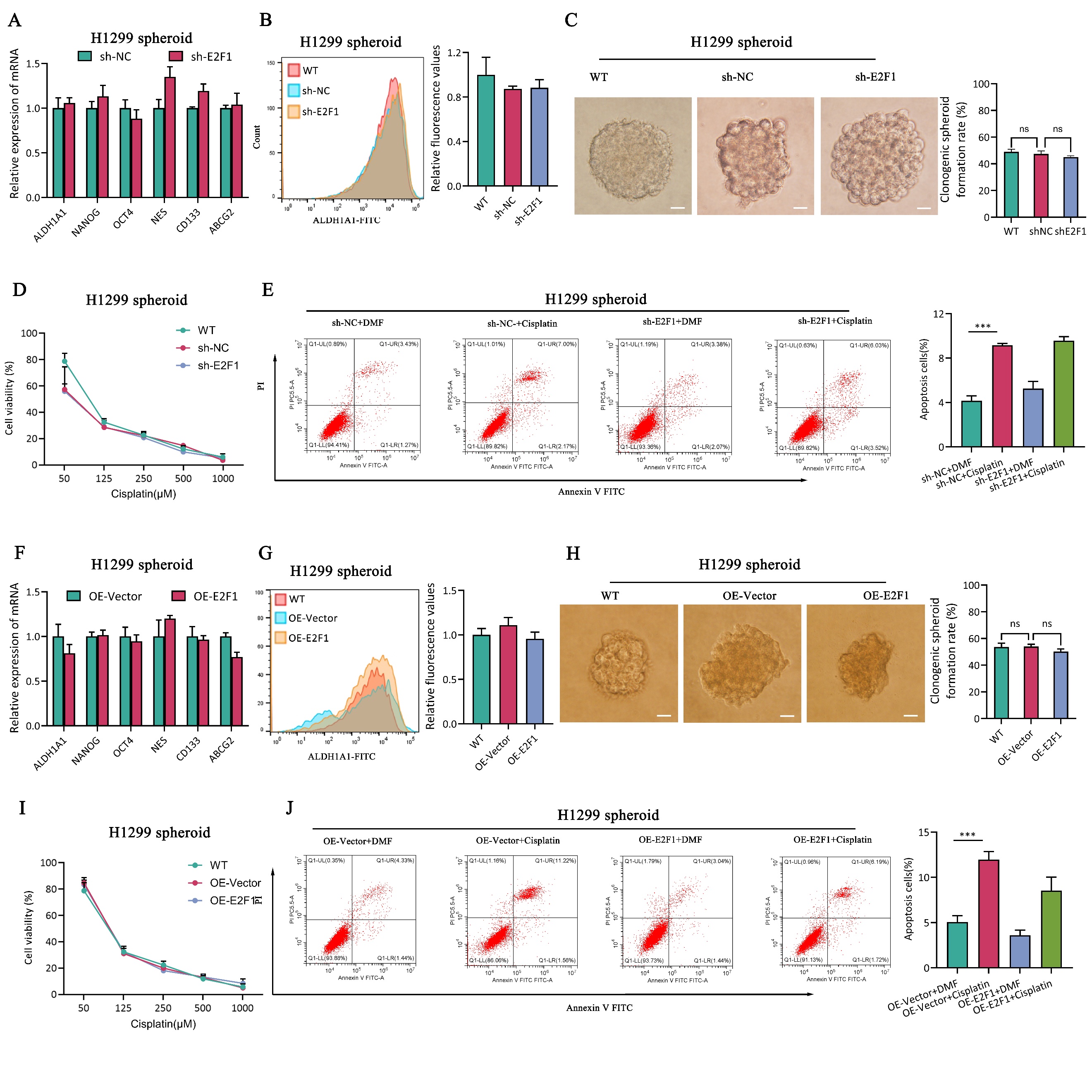


**Figure S3. No effect of E2F1 on the self-renewal and drug resistance in H1299 spheroid cells.**

A-C. The self-renewal capability of 1299 spheroid sh-NC and sh-E2F1 cells. A. mRNA abundance of CSCs marker. B. The protein expression of ALDH1A1 by flow cytometry. C. Single clone assay. D-E. The drug resistance of H1299 spheroid sh-NC and sh-E2F1 cells. D. CCK8 assay. E. The apoptosis by flow cytometry analysis. F-H. The self-renewal capability of H1299 spheroid OE-Vector and OE-E2F1 cells. F. mRNA expression of stemness genes. G. ALDH1A1 expression by flow cytometry. H. Single clone assay. I-J. The drug tolerance of H1299 spheroid OE-Vector and OE-E2F1 cells. I. CCK8. J. Flow cytometry analysis. **P* < 0.05, ***P* < 0.01, ****P* < 0.001.


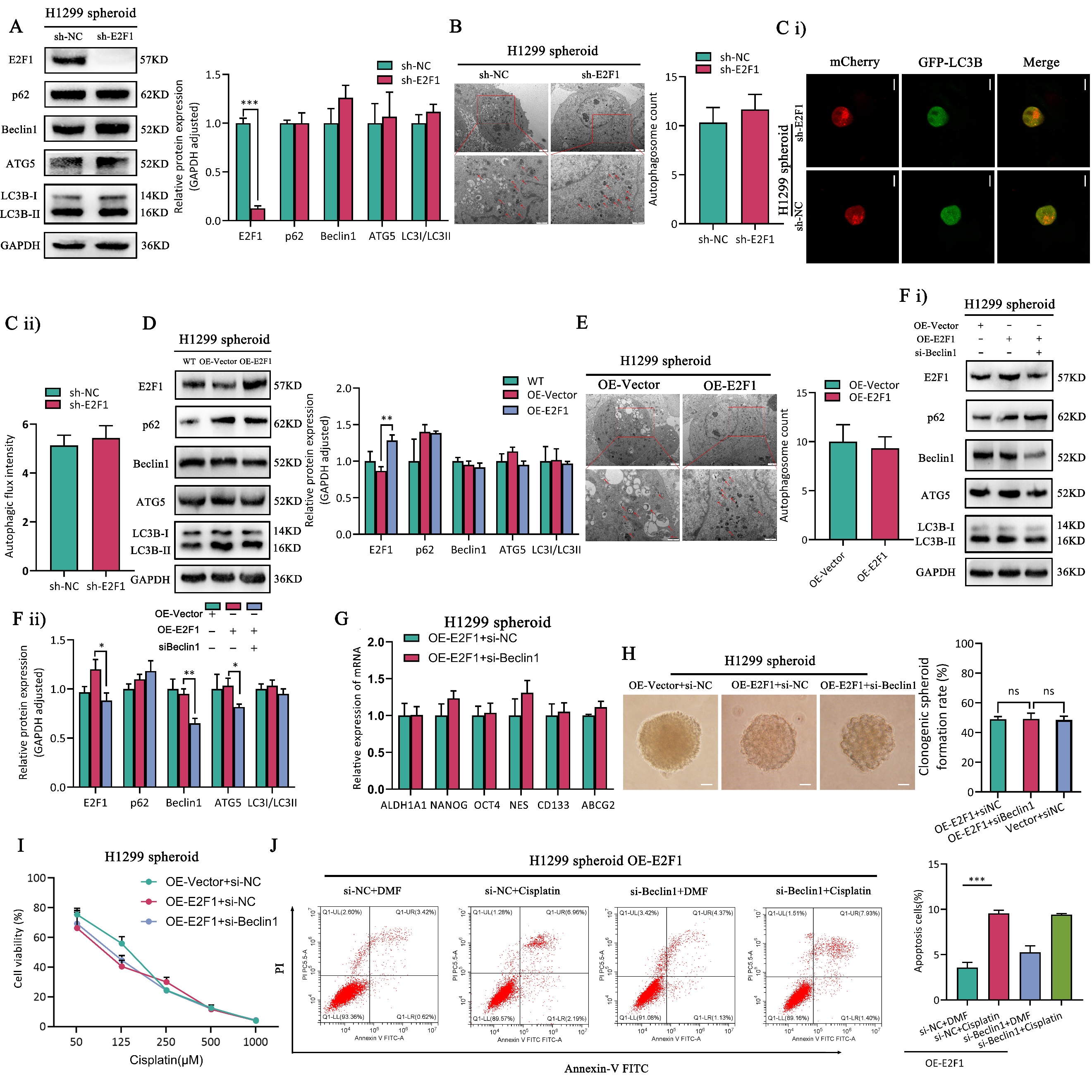


**FigureS4. Responses to self-renewal and drug resistance in H1299 spheroid cells without E2F1 regulation autophagy**

A-C. The autophagy flux of H1299 spheroid sh-NC and sh-E2F1 cells. A. Western blotting. B. TEM analysis. C. autophagic flux by confocal microscope. D-E. The autophagy flux of H1299 spheroid OE-Vector and OE-E2F1 cells. D. Western blotting. E. TEM analysis. F. The autophagy protein expression of H1299 spheroid OE-E2F1+si-NC and OE-E2F1+si-Beclin1 cells. G-H. The self-renewal ability of H1299 spheroid OE-E2F1+si-NC and OE-E2F1+si-Beclin1 cells. G. RT-qPCR. H. Single clone assay. I-J. The drug tolerance of H1299 spheroid OE-E2F1+si-NC and OE-E2F1+si-Beclin1 cells. I. CCK8 assay. J. Flow cytometry analysis. **P* < 0.05, ***P* < 0.01, ****P* < 0.001.


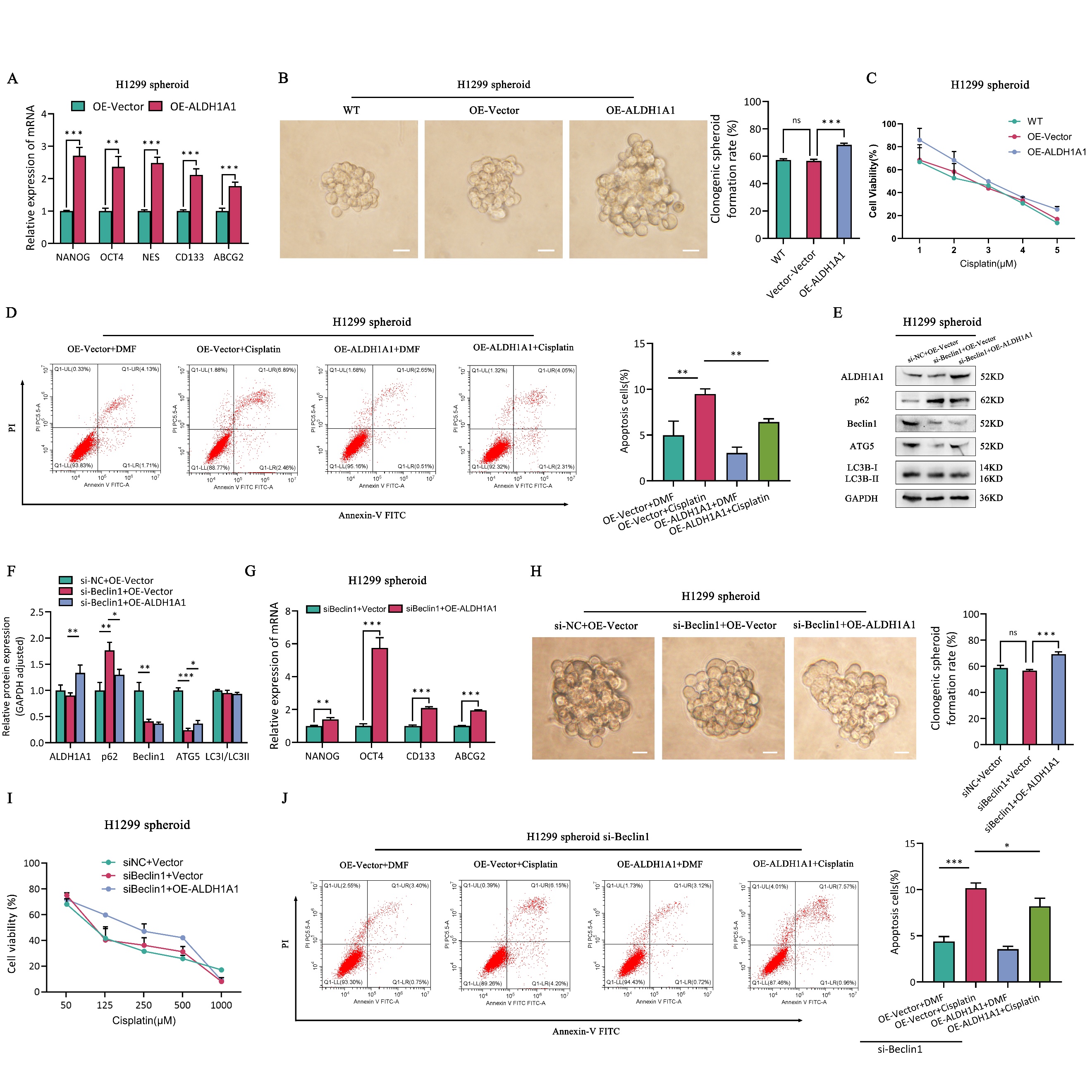


**FigureS5. ALDH1A1 enhances H1299 spheroid cells self-renewal and drug resistance.**

A-B. The self-renewal activity of H1299 spheroid OE-Vector and OE-ALDH1A1 cells. A. The stemness relative gene mRNA abundance. B. The single clone assay. C-D. The drug resistance of H1299 spheroid OE-Vector and OE-ALDH1A1 cells. C. The proliferation activity. D. Flow cytometry assay. E-F. The autophagy protein expression. G-H. The self-renewal capability of H1299 spheroid si-Beclin1+OE-Vector and si-Beclin1+OE-ALDH1A1 cells. G. RT-qPCR. H. Single clone assay. I-J. The therapy tolerance of H1299 spheroid si-Beclin1+OE-Vector and si-Beclin1+OE-ALDH1A1 cells. I. CCK8 assay. J. Cell apoptosis by flow cytometry.


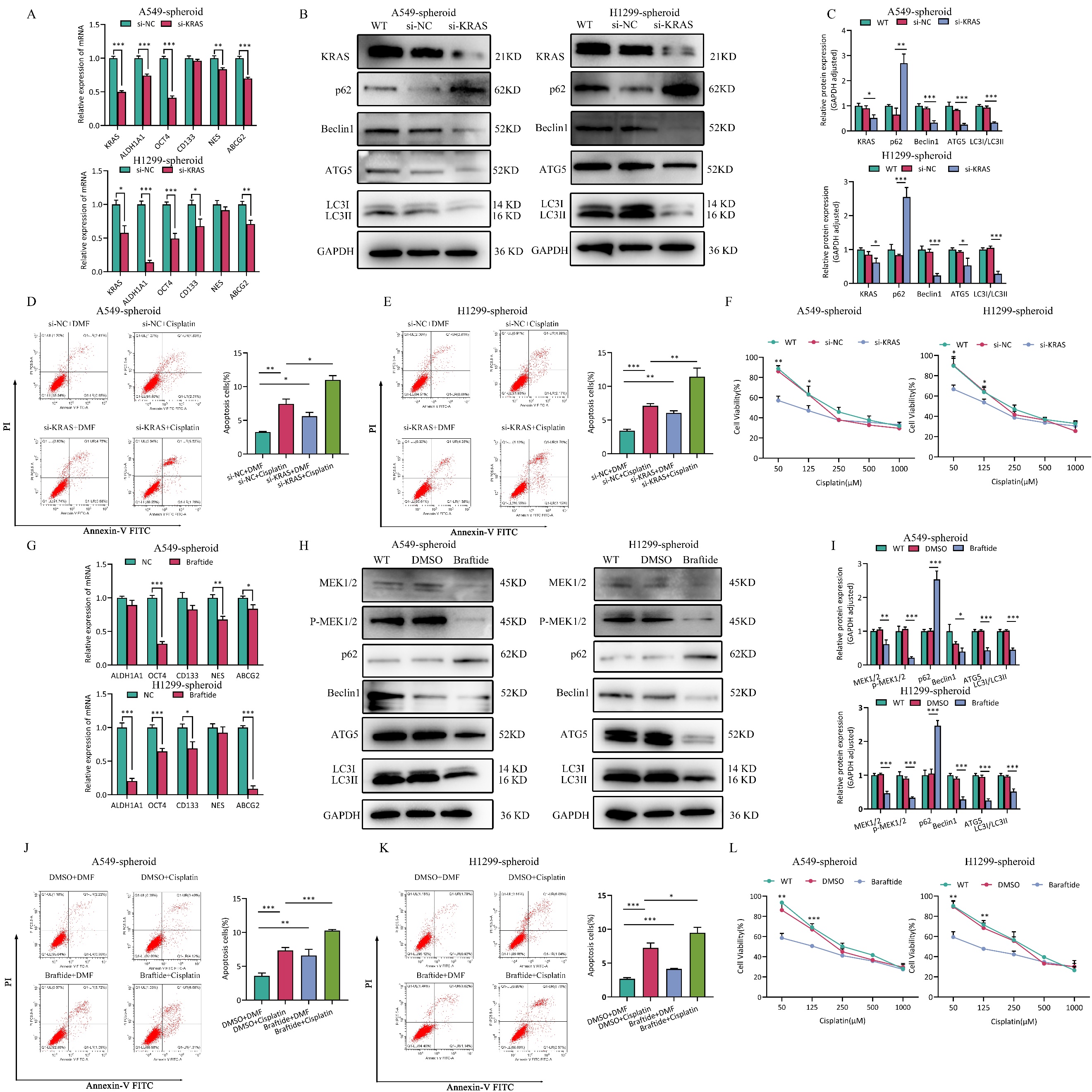


**FigureS6.** **Impact of KRAS/BRAF signaling on stemness, autophagy, and chemo-resistance in LUAD spheroids.**

A. The mRNA abundance of CSCs marker of si-NC and si-KRAS group in LUAD spheroid cells. B-C. The protein levels of p62, Beclin1, ATG5, LC3B in LUAD spheroid si-NC and si-KRAS cells assessed by western blotting. D-E. The drug resistance of LUAD spheroid si-NC and si-KRAS cells detected by flow cytometry. F. CCK8 assay. G. The mRNA expression of stemness genes in LUAD spheroid treated with inhibitor Braftide. H-I. The protein levels of p62, Beclin1, ATG5, LC3B in LUAD spheroid treated with inhibitor Braftide. J-K. The drug tolerance of LUAD spheroid treated with inhibitor Braftide. L. CCK8 assay. **P* < 0.05, ***P* < 0.01, ****P* < 0.001.


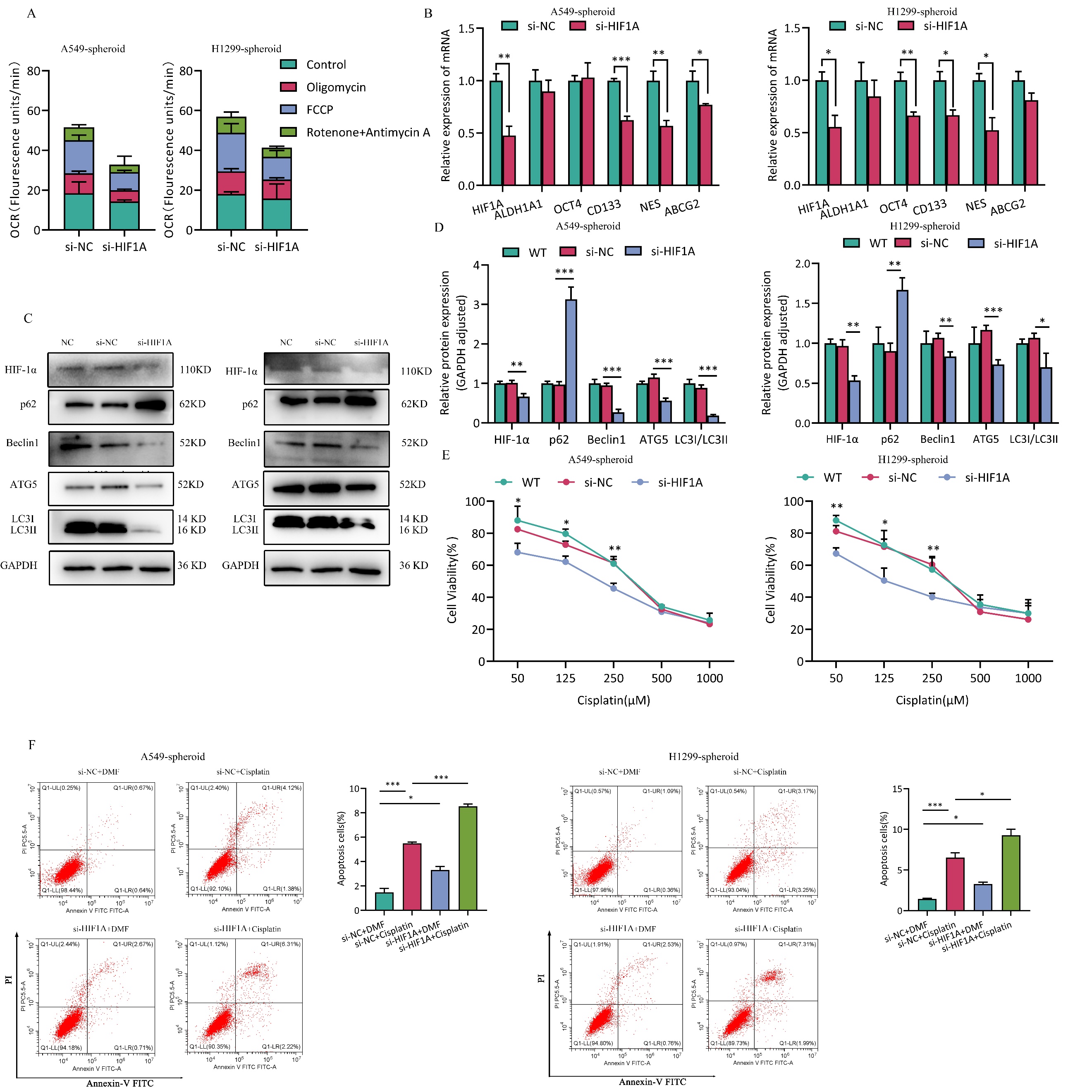


**FigureS7.** **Effect of HIF1A Knockdown on metabolism, stemness, autophagy, and drug resistance in LUAD spheroids.**

A. OCR assay of LUAD spheroid si-NC and si-HIF1A cells. B. mRNA abundance of CSCs marker of LUAD spheroid si-NC and si-HIF1A cells. C-D. The protein levels of p62, Beclin1, ATG5, LC3B in LUAD spheroid si-NC and si-HIF1A cells assessed by western blotting. E. CCK8 assay. F. The drug resistance of LUAD spheroid si-NC and si-HIF1A cells. **P* < 0.05, ***P* < 0.01, ****P* < 0.001.

**Table S1. Primers for RT‐qPCR.**

| Gene name | Forward primers | Reverse primers |
| --- | --- | --- |
| E2F1 | CACCATAGTGTCACCACCACCATC | TCTTGCTCCAGGCTGAGTAGAGAC |
| ALDH1A1 | ACGCCAGACTTACCTGTCCTACTC | TCTTGCCACTCACTGAATCATGCC |
| TP53 | GCCCATCCTCACCATCATCACAC | GCACAAACACGCACCTCAAAGC |
| NANOG | ACACTGGCTGAATCCTTCCTCTCC | ACACTGGCTGAATCCTTCCTCTCC |
| OCT4 | CCAACTCCAACCATGAGGAAGACG | CCCAGCAGCCTCAAAATCCTCTC |
| NES | AGAGCGAGCAGGAGGAGTTGG | GGAGTGGAGTCTGGAAGGGTCTC |
| ABCG2 | CGTGGCTGGTGTTGAGTGTCTG | ACCGCCTAGTGGAGCTATGAGAAG |
| CD133 | GTGGCGTGTGCGGCTATGAC | CCAACTCCAACCATGAGGAAGACG |
| ATG5 | ATCCCACAGCCAACAGATTGAAGG | TGCCTCCACCAAACCTGATTGAAG |
| LC3B | GCAGGGTAAACGGGCTGTGTG | GAGTGAGGACTTTGGGTGTGGTTC |
| Beclin1 | ACATCTGGCACAGTGGACAGTTTG | AGCATGGAGCAGCAACACAGTC |
| SQSTM1 | GCAGGGTAAACGGGCTGTGTG | GAGTGAGGACTTTGGGTGTGGTTC |
| KRAS | ACAGAGAGTGGAGGATGCTTT | TTTCACACAGCCAGGAGTCTT |
| TBP | CCGGAATCCCTATCTTTAGTCC | GCCTTTGTTGCTCTTCCAAAAT |

ALDH1A1: Aldehyde dehydrogenase 1A1. NANOG: Nanog homeobox. OCT4: octamer binding transcription 4. NES: Nestin. ABCG2: ATP-binding cassette superfamily G member 2. CD133: cluster of differentiation133. LC3B: MAP1LC3B/LC3B. SQSTM1: Sequestosome 1. TBP: TATA-binding protein.

**Table S2. siRNA sequence.**

| Name of the sequence | Sequence |
| --- | --- |
| si-NC (Beclin1) | ACGUGACACGUUCGGAGAATT |
| si-Beclin1#1 | UAUUGAUUGUGCCAAACUGTT |
| si-Beclin1#2 | AAUCUCAUUCCAUUCCACGGGTT |
| si-Beclin1#3 | UUCUGUGGACAUCAUCCUGGCTT |
| si-NC (KRAS) | UUAGGCAUUGACUUGAUUAA |
| si-KRAS | GGACUUAGCAAGAAGUUAUTT |
| si-NC (HIF1A) | AAGGCUUAUUUCGUCAUCGG |
| si-HIF1A | GUUGCCACUUCCACAUAAUTT |

siRNA: small interfering RNA.
